# FUS/circ_002136/miR-138-5p/SOX13 feedback loop regulates angiogenesis in Glioma

**DOI:** 10.1186/s13046-019-1065-7

**Published:** 2019-02-08

**Authors:** Zhenwei He, Xuelei Ruan, Xiaobai Liu, Jian Zheng, Yunhui Liu, Libo Liu, Jun Ma, Lianqi Shao, Di Wang, Shuyuan Shen, Chunqing Yang, Yixue Xue

**Affiliations:** 1 Department of Neurobiology, School of Life Sciences, China Medical University, Shenyang, 110122 People’s Republic of China; 2 Key Laboratory of Cell Biology, Ministry of Public Health of China, and Key Laboratory of Medical Cell Biology, Ministry of Education of China, China Medical University, Shenyang, 110122 People’s Republic of China; 3 Department of Neurosurgery, Shengjing Hospital of China Medical University, Shenyang, 110004 People’s Republic of China; 4 Liaoning Research Center for Translational Medicine in Nervous System Disease, Shenyang, 110004 People’s Republic of China; 5 Key Laboratory of Neuro-oncology in Liaoning Province, Shenyang, 110004 People’s Republic of China

**Keywords:** Angiogenesis, FUS; circ_002136, miR-138-5p, SOX13, SPON2

## Abstract

**Background:**

Angiogenesis plays a critical role in the progression of glioma. Previous studies have indicated that RNA-binding proteins (RBPs) interact with RNAs and participate in the regulation of the malignant behaviors of tumors. As a type of endogenous non-coding RNAs, circular RNAs (circRNAs) are abnormally expressed in various cancers and are involved in diverse tumorigeneses including angiogenesis.

**Methods:**

The expression levels of *FUS, circ_002136*, *miR-138-5p*, *SOX13,* and *SPON2* were determined using quantitative real-time PCR (qRT-PCR) and western blot. Transient cell transfection was performed using the Lipofectamine 3000 reagent. The RNA-binding protein immunoprecipitation (RNA-IP) and the RNA pull-down assays were used to detect the interaction between *FUS* and *circ_002136*. The dual-luciferase reporter assay system was performed to detect the binding sites of *circ_002136* and *miR-138-5p*, *miR-138-5p* and *SOX13*. The chromatin immunoprecipitation (ChIP) assays were used to examine the interactions between transcription factor *SOX13* and its target proteins .

**Results:**

We demonstrated that down-regulation of *FUS* or *circ_002136* dramatically inhibited the viability, migration and tube formation of U87 glioma-exposed endothelial cells (GECs). *MiR-138-5p* was down-regulated in GECs and *circ_002136* functionally targeted *miR-138-5p* in an RNA-induced silencing complex (RISC). Inhibition of *circ_002136,* combined with the restoration of *miR-138-5p,* robustly reduced the angiogenesis of GECs. As a target gene of *miR-138-5p*, *SOX13* was overexpressed in GECs and was proved to be involved in *circ_002136* and *miR-138-5p*-mediated angiogenesis in gliomas. In addition, we found that *SOX13* was directly associated with and activated the *SPON2* promoter, thereby up-regulating the expression of *SPON2* at the transcriptional level. Knockdown of *SPON2* suppressed the angiogenesis in GECs. More important, *SOX13* activated the *FUS* promoter and increased its expression, forming a feedback loop.

**Conclusion:**

Our data suggests that the feedback loop of *FUS/circ_002136/miR-138-5p/SOX13* played a crucial role in the regulation of angiogenesis in glioma. This also provides a potential target and an alternative strategy for combined glioma therapy.

**Electronic supplementary material:**

The online version of this article (10.1186/s13046-019-1065-7) contains supplementary material, which is available to authorized users.

## Background

Malignant glioma is the most common primary tumor in the human central nervous system. Despite many advances in surgical techniques, chemotherapeutics and radiation therapy, the prognosis of patients with malignant gliomas remains obstinately poor. Glioma progression depends on tumor blood vessel growth. Glioma cells secrete vascular endothelial growth factor and other pro-angiogenic factors to promote the growth of vascular endothelial cells. Moreover, glial vascular endothelial cells also secrete a variety of factors that promote tumor growth. The interactions of these secreted factors can promote glioma growth [1]. Therefore, anti-angiogenesis therapeutics are considered as important treatments for malignant glioma, and an in-depth study of angiogenesis in glioma is in need.

The *FUS* (Fused in sarcoma) gene is located at chromosome 16p11.2 and consists of 15 exons encoding a protein of 526 amino acids belonging to the FET (FUS/EWS/TAF15) protein family. As a DNA/RNA-binding protein with a gene regulation function, it is involved in regulating intracellular RNA transport, mRNA synthesis, alternative splicing, and polyadenylation site selection [2]. It has been found that *FUS* mRNA or protein expression is up-regulated in liposarcoma [3], breast cancer [4], cervical cancer [5], and other cells. *FUS* can promote the malignant progression of non-small cell lung cancer [6]. Silencing of the *FUS* expression inhibits the proliferation and migration of neuroblastoma cells and increased their chemosensitivity to cisplatin [7]. A recent study has confirmed that *FUS* regulates the expression of 19 circRNAs, including *circ_3279* and *circ_5306,* via binding to introns flanking the splicing junction [8]. But the function of *FUS* in vascular endothelial cells has not yet been reported.

CircRNA is a non-coding RNA with a covalent loop structure, which can perform biological functions via various modes of regulation. For example, circRNAs can affect gene expression or transcription by regulating transcription and alternative splicing [9]. CircRNAs may also act as molecular sponges of microRNAs (miRNAs) or competitive endogenous RNAs to regulate translation of the target genes [10]. Previous studies have shown that circRNAs play regulatory roles in the malignant biological behavior of glioma cells. For example, *circ-TTBK2* and *circ-HIPK3* promote malignant progression of glioma cells [11, 12]. *Hsa_circ_0000177* is significantly up-regulated in human glioma cells, promoting cell proliferation, invasion in vitro, and growth of glioma in vivo [13]. Human *circ_002136* (*hsa_circ_0000005*, *hsa_circCDK11A_001*) formed through the looping of linear *CDK11A-VT1* (*Homo sapiens* cyclin-dependent kinase 11A transcript variant 1; GenBank: NM_024011) is located at chromosome 1 and is 49,639 bps in length. To date, the function and mechanism of *circ_002136* have not been clarified.

MiRNAs regulate the expression of target genes at the post-transcriptional level via binding to the 3′-untranslated region (3’-UTR) of the target genes. Studies have shown that over-expression of *miR-138-5p* suppresses tumor cell proliferation, invasiveness and induces apoptosis in pancreatic cancer [14] and bladder cancer [15]. The *SOX13* gene is located at chromosome 1q31.3–32.1 and consists of 14 exons belonging to the SOX gene family (Sex-related region Y, Sry-related high-mobility group box). It has been found that *SOX13* is highly expressed in oligodendroglioma [16] and can regulate angiogenesis [17]. However, the roles of *miR-138-5p* and *SOX13* in glial vascular endothelial cells and their potential molecular mechanisms remain poorly defined.

*SPON2* (*Spondin 2*, *Mindin*) is a member of the Mindin F-Spondin superfamily, which encodes secreted proteins and extracellular matrix proteins. *SPON2* is highly expressed in ovarian cancer [18] and breast cancer [19]. In patients with hepatocellular carcinoma, the expression of *SPON2* is positively correlated with prognosis [20]. These suggest that *SPON2* plays an essential role in the development of various tumors. However, the function of *SPON2* in vascular endothelial cells of glioma is still unknown.

In our study, we first identified the endogenous expressions of *FUS*, *circ_002136*, *miR-138-5p*, *SOX13,* and *SPON2* in GECs, and explored possible regulatory interactions among the factors mentioned above and their impacts on the GEC angiogenesis. Our results provide a new insight into the mechanism of angiogenesis in glioma as well as new strategies for anti-angiogenesis treatment of glioma.

## Methods

### Cell culture and the preparation of glioblastoma (GBM) cell-conditioned medium

The immortalized human cerebral microvascular endothelial cell (EC) line hCMEC/D3 was provided by Dr. Couraud from the Institut Cochin, Paris, France. Cells were cultured in endothelial basal medium (EBM-2) (Lonza, Walkersville, MD, USA), supplemented with 5% fetal bovine serum (FBS) “Gold” (PAA Laboratories, Pasching, Austria), 1% penicillin-streptomycin (Life Technologies, Paisley, UK), 1% chemically defined lipid concentrate (Life Technologies), 1 ng/mL human basic fibroblast growth factor (bFGF) (Sigma-Aldrich, Beijing, China), 1.4 μM hydrocortisone, 5 μg/mL ascorbic acid (Sigma-Aldrich), and 10 mM N-2-hydroxyethylpiperazine-N-ethane-sulphonic acid (HEPES) (PAA Laboratories). ECs were limited with the passage below 30. The human glioblastoma cell line (U87MG) and human embryonic kidney 293 T (HEK293T) cell line were purchased from the Shanghai Institutes for Biological Sciences Cell Resource Center (Shanghai, China) and were cultured in high-glucose Dulbecco’s modified Eagle medium (DMEM, GIBCO, Carlsbad, CA, USA), supplemented with 10% FBS. All cells were maintained in a humidified incubator at 37 °C with 5% CO_2_.

GBM cell-conditioned medium was collected from the human glioblastoma cell line U87 plated in 100-mm-diameter Petri dishes. Cells that had grown to near confluency were washed twice with serum-free medium and incubated in serum-free EBM-2 medium for 24 h. The supernatant was harvested, centrifuged at 2000×g at 4 °C for 10 min and supplemented with 5% FBS, 1% penicillin-streptomycin, 1% chemically defined lipid concentrate, 1 ng/ml bFGF, 1.4 μM hydrocortisone, 5 μg/ml ascorbic acid, 10 mM HEPES, and stored at 4 °C. The GBM cell-conditioned medium was used to culture human cerebral microvascular endothelial cells (hCMEC/D3) for 24 h to produce GECs.

### Quantitative real-time PCR

Total RNAs were extracted from ECs and GECs with Trizol reagent (Life Technologies, Carlsbad, CA, USA) according to the manufacturer’s description. RNA concentration and quality were determined for each sample with a Nanodrop Spectrophotometer (ND-100; Thermo Fisher Scientific, Waltham, MA, USA) using the 260/280 nm ratio. One Step SYBR PrimeScript RT-PCR Kit (Takara Biomedical Technology, Dalian, China) was used to qualify the expression levels of *FUS* (NM_004960.3), *circ_002136*(NM_024011), *CDK11A*(NM_024011.3), *SOX13*(NM_005686.2), and *SPON2*(NM_012445.3). In addition, RNase R was used to confirm the existence of *circ_002136* and eliminate the influence of linear RNAs. Glyceraldehyde 3-phosphate dehydrogenase (GAPDH) was used as an endogenous control. The expression levels of *miR-138-5p* (NR_029700.1) were detected using the TaqMan MicroRNA Reverse Transcription Kit (Applied Biosystems, Foster City, CA, USA) and TaqMan Universal Master Mix II (Life Technologies). The U6 housekeeping gene was included as an endogenous control. All qRT-PCR reactions were performed using the 7500 Fast RT-PCR System (Applied Biosystems). The relative quantification 2-^ΔΔCt^ method was applied to calculate the gene expression values. Primers and probes used in this study are shown in Table 1.

**Table 1:** Primers and probes used for RT-qPCR

Primer or Probe	Gene	Sequence (5′- > 3′) or Assay ID
Primer	FUS	F: GCCAAGATCAATCCTCCATGAGTAGTG
		R: TCCACGGTCCTGCTGTCCATAG
	circ_002136	F: CTTTCCGAGACATTTGCTGG
		R: CATGGAGATCACAATAAGGAACTC
		P:FAM + TCTTCTTCTCCTCTGTCTTCC+MGB
	GAPDH	F: GGACCTGACCTGCCGTCTAG
		R: TAGCCCAGGATGCCCTTGAG
		P:FAM + CCTCCGACGCCTGCTTCACCACCT+Eclipse
	CDK11A	F: AGAGGAAGAGGAGGAGGAGGAGAC
		R: CGAACCGTGACTCTGGAACAACC
	SOX13	F: CTGGACTTCAACCGAAATTTGA
		R: GTTCCTTCCTAGAAACCTCTCC
	SPON2	F: GATTGTAGACAGCGCCTCAGTTCC
		R: GACGCACTCAGCCTCTTCTTCG
Probe	MiR-138-5p U6	002284(Applied Biosystems)
		001973(Applied Biosystems)

### Western blot assay

Total protein was extracted from cells using ice-cold radioimmunoprecipitation assay (RIPA) buffer (Beyotime Institute of Biotechnology, Jiangsu, China) supplemented with protease inhibitors (10 mg/mL aprotinin, 10 mg/mL phenyl-methylsulfonyl fluoride [PMSF], and 50 mM sodium orthovanadate). The BCA protein assay kit (Beyotime Institute of Biotechnology) was used to determine the protein concentration of the supernatant. Equal amounts of protein samples (50 μg) were separated by sodium dodecyl sulfate-polyacrylamide gel electrophoresis (SDS-PAGE) and electrically transferred onto polyvinylidene difluoride (PVDF) membrane (Millipore, Shanghai, China). Non-specific binding was blocked by incubation with 5% fat-free milk in Tris-buffered saline containing 0.1% Tween-20 (TBST) at room temperature for two hours.

The membranes were subsequently incubated with primary antibodies as follows: *FUS* (1:2000; Proteintech, Chicago, IL, USA), *SOX13* (1:500; Proteintech), *SPON2* (1:1000; Affinity, Cincinnati, OH, USA), and GAPDH (1:10,000; Proteintech) at 4 °C overnight. The membranes were washed and incubated with HRP-conjugated secondary antibodies (Santa Cruz Biotechnology, Santa Cruz, CA, USA), diluted at 1:5000 at room temperature for two hours. Immunoblots were visualized using an enhanced chemiluminescence kit (ECL; Santa Cruz Biotechnology) and detected by ECL Detection Systems (Thermo Scientific, Beijing, China) and then scanned using Chemi Imager 5500 V2.03 software. The relative integrated density values (IDVs) were calculated by FluorChem 2.0 software and then normalized to that of GAPDH.

### Plasmid construction and cell transfection

Short-hairpin RNAs (shRNAs) directed against human *FUS, circ_002136, CDK11A, SOX13,* and *SPON2* gene were constructed in pGPU6/GFP/Neo vector (GenePharma, Shanghai, China) to generate silencing plasmids. The human *SOX13* gene coding sequence was ligated into the pIRES2-EGFP vector (GenScript, Piscataway, NJ, USA) to construct the *SOX13*-over-expression plasmid. pGPU6/GFP/Neo and pIRES2-EGFP empty vectors without targeting sequences were used as negative controls (NCs). GECs were seeded into 24-well plates and transfected with the plasmids via Opti-MEM I and Lipofectamine LTX and Plus Reagents (Life Technologies) when they reached approximately 80% confluence. The stable transfected cell lines were created by selection on culture medium containing 0.4 mg/mL geneticin (G418) (Sigma-Aldrich, St. Louis, MO, USA). G418-resistant cell clones were established after approximately four weeks of use. For co-transfection of *FUS* (−) and *circ_002136* (−), cells with stably knocked down *FUS* were transfected with pGPU6/*circ_002136* (−)/Blasticidin. G418 and Blasticidin dual-resistant clone were selected. qRT-PCR was then performed to measure the transfected efficiencies.

Furthermore, *miR-138-5p* agomir [*miR-138-5p* (+); GenePharma], *miR-138-5p* antagomir [*miR-138-5p* (−)], and their respective NCs were transiently transfected into GECs using Lipofectamine 3000 Reagents (Life Technologies). For co-transfection of *circ_002136* (−) and *miR-138-5p* agomir/ antagomir or *SOX13* (+) and *miR-138-5p* agomir, cells with stably knocked down *circ_002136* or overexpressed *SOX13*, or their respective NCs, were transiently transfected with *miR-138-5p* agomir/antagomir or their NCs. All transient-transfected cells were harvested after 48 h. The sequences of all shRNA templates are shown in Table 2. The transfection efficiencies are shown in Additional file 1: Figure S1.

**Table 2:** Sequences of shRNA template

Gene		Sequence(5′- > 3′)
FUS	Sense	CACCGCCCTACGGACAGCAGAGTTTCAAGAGAACTCTGCTGTCCGTAGGGTTTTTTG
	Antisense	CGGGATGCCTGTCGTCTCAAAGTTCTCTTGAGACGACAGGCATCCCAAAAAACCTAG
Circ_002136	Sense	CACCGCTATGGAAGACAGAGGAGAAGTTCAAGAGACTTCTCCTCTGTCTTCCATAGTTTTTTG
	Antisense	CGATACCTTCTGTCTCCTCTTCAAGTTCTCTGAAGAGGAGACAGAAGGTATCAAAAAACCTAG
CDK11A	Sense	CACCAGAUCUACAUCGUGAUGAATTTTCAAGAGAUUCAUCACGAUGUAGAUCUTGTTTTTTG
	Antisense	GATCCAAAAAAAGAUCUACAUCGUGAUGAATTTCTCTTGAAUUCAUCACGAUGUAGAUCUTG
SOX13	Sense	CACCGGAAGATCCTGCAAGCCTTCCTTCAAGAGAGGAAGGCTTGCAGGATCTTCCTTTTTTG
	Antisense	GATCCAAAAAAGGAAGATCCTGCAAGCCTTCCTCTCTTGAAGGAAGGCTTGCAGGATCTTCC
SPON2	Sense	CACCGGGCGCTGATGAAGGAGATCGTTCAAGAGACGATCTCCTTCATCAGCGCCCTTTTTTG
	Antisense	CCCGCGACTACTTCCTCTAGCAAGTTCTCTGCTAGAGGAAGTAGTCGCGGAAAAAACCTAG
NC	Sense	CACCGTTCTCCGAACGTGTCACGTCAAGAGATTACGTGACACGTTCGGAGAATTTTTTG
	Antisense	GATCCAAAAAATTCTCCGAACGTGTCACGTAATCTCTTGACGTGACACGTTCGGAGAAC

### Cell viability assay

Cell Counting Kit-8 (CCK-8, Beyotime Institute of Biotechnology) assay was conducted to determine the viability of GECs. Cells were plated in 96-well plates at a density of 2000 cells/well and incubated in GBM cell-conditioned medium for 24 h. Each well was incubated with 10 μL CCK-8 solution at 37 °C for 2 h. Optical density values were evaluated at 450 nm using the SpectraMax M5 microplate reader (Molecular Devices, San Jose, CA, USA).

### Cell migration assay

The migration ability of GECs was assessed using a 6.5 mm Transwell with 8.0 μm Pore Polycarbonate Membrane Insert (#3422, Corning, NY, USA). The upper chamber was used to incubate cells resuspended in 200 μL serum-free medium at a density of 5 × 10^5^ cells/mL. The lower chamber was filled with 600 μL GBM cell-conditioned medium.

After incubation at 37 °C for 48 h, non-migrated cells on the top surface of the membrane were carefully removed. Migrated cells on the lower surface of the membrane were fixed with methanol and glacial acetic acid at the ratio of 3:1 and stained with 10% Giemsa solution (Dinguo, Beijing, China). Then, images of the stained cells were taken with an inverted microscope, and the cell numbers in five randomly selected fields were counted for statistical analysis in each well.

### Tube formation assay

Matrigel assay was performed to evaluate the tube formation of GECs. Pre-chilled 96-well plates were coated with 100 μL Matrigel (BD Biosciences, Bedford, MA, USA) per well and incubated to polymerize at 37 °C for 30 min. ECs were resuspended in 100 μL GBM cell-conditioned medium and seeded onto the surface of the polymerized Matrigel at a density of 4 × 10^5^ cells/mL, followed by incubation at 37 °C for 6 h. Olympus DP71 microscopy (Olympus, Tokyo, Japan) was used to acquire three or more images at random from each culture, and ImageJ software was used to measure the total tubule length and the number of branches.

### Reporter vector construction and luciferase reporter assays

The putative binding sequences and mutant sequences of *miR-138-5p* in *circ_002136* and *SOX13* 3′-UTR were amplified by PCR and cloned downstream of the pmirGLO Dual-luciferase miRNA Target Expression Vector (Promega, Madison, WI, USA) to construct a dual-luciferase reporter vector (*circ_002136*Wt/*circ_002136*Mut and *SOX13*–3’-UTR-Wt/*SOX13*–3’-UTR-Mut, GenePharma). *Circ_002136*Wt/*circ_002136*Mut or *SOX13*–3’-UTR-Wt/*SOX13*–3’-UTR-Mut dual-luciferase vectors and *miR-138-5p* agomir (or agomir NC) plasmids were co-transfected into HEK293T cells using Lipofectamine 3000. The dual-luciferase activity was measured 48 h after transfection. The Dual-Luciferase Reporter Assay System (Promega) was used to analyse luciferase activity. Relative luciferase activity was expressed as the ratio of firefly luciferase activity to Renilla luciferase activity.

For the *SOX13*-*SPON2* reporter constructs, the *SPON2* promoter region (− 1, 000 to + 200 bp) was amplified from human genomic DNA by PCR and then subcloned into pGL3-Basic-Luciferase vector (Promega) containing a firefly luciferase reporter gene, yielding the wide-type plasmid (*SPON2*-Wt). In addition, to test the binding specificity, corresponding mutants of putative *SOX13* binding sites were created to form the reporter vector *SPON2*-mutated-types (*SPON2*-Mut1, *SPON2*-Mut2, *SPON2*-Mut3 and *SPON2*-Mut4) (GenePharma). The human full-length *SOX13* gene was constructed into pEX3 (pGCMV/MCS/Neo) plasmid vector (GenePharma). HEK293T cells were co-transfected with pGL3 vector (either with wide-type promoter regions or mutated promoter regions) and pEX3-*SOX13* (or pEX3 empty vector) using Lipofectamine 3000. The promoter activity of constructed plasmid was normalized with the co-transfected reference vector (pRL-TK) and expressed as relative to the activity of pEX3 empty vector, which the activity set to 1.

### RNA pull-down assay

The interaction between RBP *FUS* and *circ_002136* introns was detected using Pierce Magnetic RNA-Protein Pull-Down Kit (ThermoFisher) according to the manufacturer’s protocols. In brief, *circ_002136* transcripts were transcribed using T7 RNA polymerase (Ambion Life, Taoyuan, Taiwan). Biotin RNA Labeling Mix (Ambion Life) was used to biotin-label the purified RNAs, and then the positive control (Input), negative control (Antisense RNA), and biotinylated RNAs were mixed and co-incubated with proteins extracted from ECs at room temperature. Magnetic beads were added to prepare a probe–magnetic bead complex. Then the bead complex was washed with Handee spin columns and boiled in SDS buffer. Finally, retrieved proteins were detected by western blot, including GAPDH as a control.

### RNA-binding protein immunoprecipitation assay

GECs lysates from different groups were incubated with RIP buffer containing magnetic beads conjugated with anti-human argonaute 2 (Ago2) antibodies (Millipore, Billerica, MA, USA). Normal mouse IgG (Millipore) was used as NCs. The samples were incubated with Proteinase K, and then immunoprecipitated RNAs were isolated. The RNA concentration was measured using a spectrophotometer (NanoDrop, ThermoScientific), and the RNA quality was assessed using a bioanalyzer (Agilent, Santa Clara, CA, USA). Purified RNAs were extracted and applied in qPCR for reverse transcription analysis.

### ChIP assay

ChIP assays were performed using the Simple ChIP Enzymatic Chromatin IP Kit (Cell Signaling Technology, Danvers, MA, USA) according to the manufacturer’s protocol. Briefly, GECs were crosslinked with EBM-2 containing 1% formaldehyde and collected in a lysis buffer containing 1% PMSF. Chromatin was digested by micrococcal nuclease, and 2% aliquots of lysate were used as an input control. Lysates were incubated with 3 μg anti- *SOX13* antibody (Proteintech) or normal rabbit IgG, followed by immunoprecipitation with protein G agarose beads and incubation at 4 °C overnight with gentle shaking. DNA crosslink was reversed by the addition of 5 mol/L NaCl and Proteinase K at 65 °C for 2 h, and finally, DNA was purified. Immunoprecipitated DNA was amplified by PCR using specific primers. Primers used for ChIP PCR are shown in Table 3.

**Table 3:** Primers used for ChIP experiments

Gene	Binding site or Control	Sequence (5′- > 3′)	Product size (bp)	Annealing temperature (°C)
SPON2	PCR1	F:TTTACCGAGTGCTAGAGCCG	165	59.6
		R: AGGCTGCTGTGGCTGTTT		
	PCR2	F: CTTACGACGCAGGGTCTGG	237	59.8
		R:CGGCTCTAGCACTCGGTAAA		
	PCR3	F:ACCCAAGAAAATCAGCCAAAGC	211	60.2
		R:TCACTGTGGAATCGCGTGAG		
	PCR4	F:GTGCCCAGCATCTATTCTGGT	207	59.9
		R: CTACAGCGTCCCACAGACC		
FUS	PCR1	F:AGTGTTTTGCAGTTACAAGACCTG	97	59.8
		R:GGAAAGTGAGACTCAGAGACCC		
	PCR2	F: CGTCTTGGCTCACTGCAACT	167	60.5
		R:GTCAGGAGTTCGAGACCAGC		

### In vivo Matrigel plug assay

Four-week-old male BALB/C athymic nude mice were purchased from the Vital River Laboratory Animal Technology Co., Ltd. (Beijing, China). The animals were fed with autoclaved food and water during the experiment. All animal procedures were performed in strict accordance with the protocol approved by the Administrative Panel on Laboratory Animal Care of China Medical University (Shenyang, China). In brief, 3 × 10^6^ GECs resuspended in 400 μL solution containing 80% Matrigel were subcutaneously injected. Plugs were harvested after 4 days and then weighed, photographed, and dispersed in 400 μL PBS (with overnight incubation at 4 °C) to collect the hemoglobin. Hemoglobin content was measured using Drabkin’s reagent solution (Sigma-Aldrich) according to the manufacturer’s instructions.

### Statistical analysis

Quantitative data were presented as mean ± standard deviation (SD). GraphPad Prism v5.01 (GraphPad, La Jolla, CA, USA) software was used for statistical analysis. Student’s t-test (two-tailed) or one-way ANOVA, followed by Bonferroni’s post-hoc test, was employed to evaluate all statistical analyses. Differences were considered statistically significant when *P* < 0.05.

## Results

### *FUS* is up-regulated in GECs; *FUS* silencing suppresses angiogenesis of glioma; *FUS* binds to and promotes the production of *circ_002136* to regulate glioma angiogenesis

**Fig. 1:**
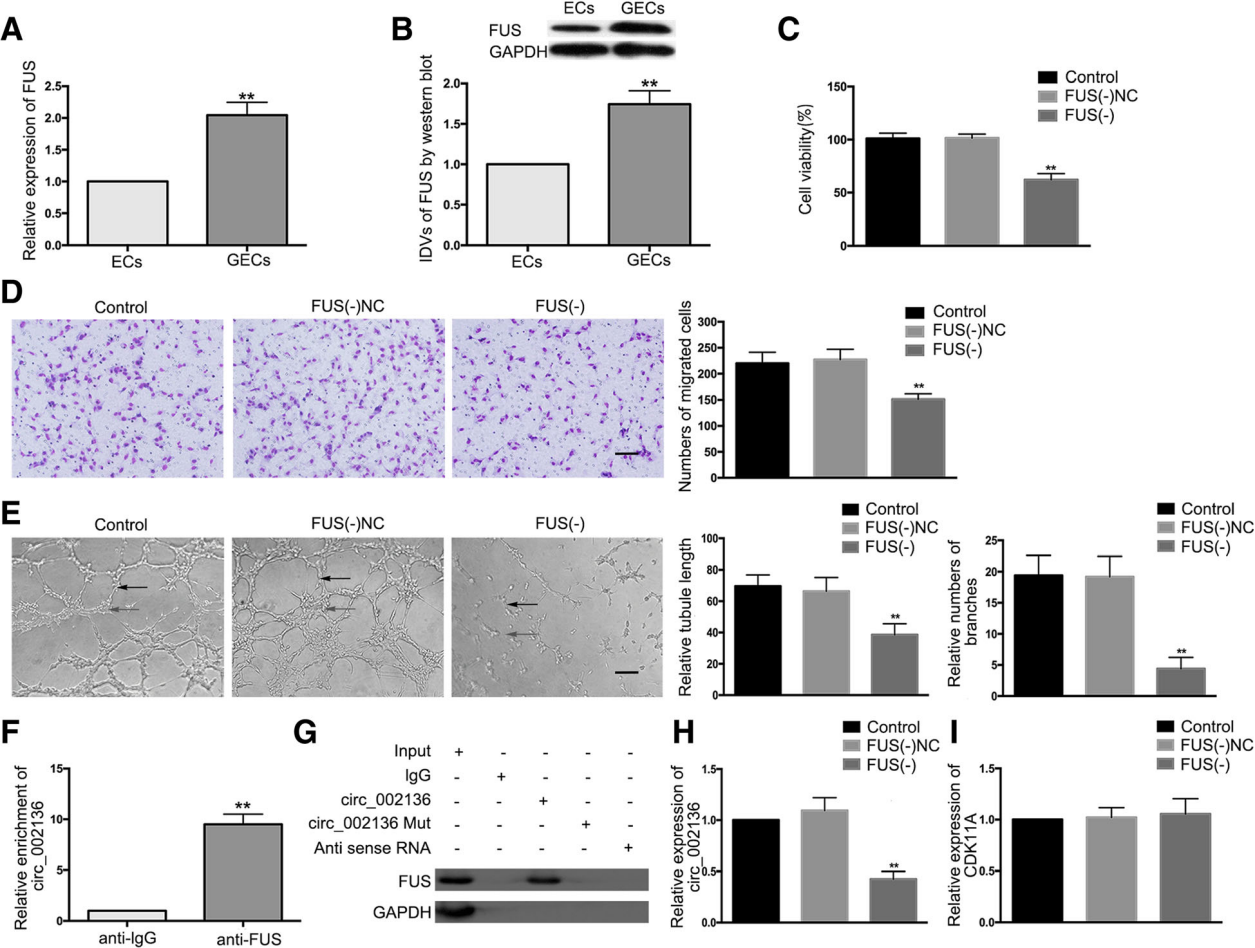
Knockdown of *FUS* suppressed GEC angiogenesis in U87 glioma. (**a**-**b**) The mRNA and protein expression levels of *FUS* in ECs and GECs were evaluated by qRT-PCR and western blot. GAPDH was used as an endogenous control. IDVs represents integrated density values. Data are presented as the means ± SD (*n* = 5, each group). ^**^P < 0.01 vs. ECs group. (**c**) The effects of *FUS* knockdown on GEC viability were detected by the CCK-8 assay. Data are presented as the means ± SD (*n* = 5, each group). ^**^P < 0.01 vs. *FUS* (−) NC group. (**d**) The effects of *FUS* knockdown on GECs were detected by the transwell assay. Data are presented as the means ± SD (*n* = 5, each group). ^**^P < 0.01 vs. *FUS* (−) NC group. The scale bar represents 100 μm. (**e**) The effects of *FUS* knockdown on GECs tube formation were measured by the Matrigel tube formation assay (black arrow, tube structures; grey arrow, tube branches). Data are presented as the means ± SD (*n* = 5, each group). ^**^P < 0.01 vs. *FUS* (−) NC group. The scale bar represents 100 μm. (**f**) The relative enrichment of *circ_002136* in anti-IgG and anti-*FUS* was detected by an RNA-IP assay. Data are presented as the means ± SD (n = 3, each group). ^**^P < 0.01 vs. anti-IgG group. (**g**) *FUS* and GAPDH protein levels immunoprecipitation with *circ_002136* were evaluated by western blot. The expression levels of *FUS* and GAPDH proteins are shown. (**h**) The effects of *FUS* knockdown on *circ_002136* expression levels were detected by qRT-PCR. Data are presented as the means ± SD (*n* = 5, each group). ^**^P < 0.01 vs. *FUS* (−) NC group. (**i**) The effects of *FUS* knockdown on *CDK11A* expression levels were detected by qRT-PCR. Data are presented as the means ± SD (*n* = 5, each group)

This study first examined the expression of *FUS* in ECs and GECs. The results showed that the mRNA and protein expression levels of *FUS* in GECs were significantly higher than those in ECs (Fig. 1A, B). The shRNA of *FUS* was then transfected into GECs to silence *FUS*, and the transfection efficiency was evaluated by western blot (Additional file 1: Figure S1A). The cell viability of GECs was detected by the CCK8 assay. The results showed that, compared to the *FUS*(˗)NC group, cell viability in the *FUS*(−) group was significantly reduced (Fig. 1C). The GEC cell migration assay showed that compared to the *FUS*(−)NC group, the migration capability of the *FUS*(−) group was significantly lower (Fig. 1D). The results of the Matrigel three-dimensional angiogenesis assay showed that compared to the *FUS*(−)NC group, relative tubule length and relative numbers of branches in the *FUS*(−) group were significantly lower (Fig. 1E).

RNA-IP assay was performed to investigate whether binding occurred between *FUS* and *circ_002136*. The relative enrichment of *circ_002136* in the *FUS* co-precipitation group was significantly increased compared to that in the IgG immunoprecipitation group (Fig. 1F). The results of RNA pull-down assays demonstrated that *FUS* combined with *circ_002136* and promoted the generation of *circ_002136* (Fig. 1G). The expression of *circ_002136* in GECs with stable knockdown of *FUS* was then further examined. The results showed that compared to the *FUS*(−)NC group, the expression of *circ_002136* in the *FUS*(−) group was significantly reduced (Fig. 1H). The expression of linear *CDK11A*, the *circ_002136* parental gene, was also detected in GECs with stable knockdown of *FUS*. The results showed no significant difference in the expression of *CDK11A* in the *FUS*(−) group compared to the *FUS*(−)NC group (Fig. 1I).

Furthermore, *circ_002136* was silenced in GECs stably transfected with the *FUS* silencing vector. Rescue experiments showed that the expression of *miR-138-5p* was significantly increased both in the *FUS*(−) + *circ_002136*(−)NC and the *FUS*(−)NC + *circ_002136*(−) groups compared to the *FUS*(−)NC + *circ_002136*(−)NC group. Compared to the *FUS*(−) + *circ_002136*(−)NC group, the expression of *miR-138-5p* was further significantly increased in the *FUS*(−) + *circ_002136*(−) group (Additional file 2: Figure S2A). However, the mRNA and protein expression levels of *SOX13* and *SPON2* were significantly decreased both in the *FUS*(−) + *circ_002136*(−)NC and *FUS*(−)NC + *circ_002136*(−) groups compared to the *FUS*(−)NC + *circ_002136*(−)NC group. In comparison with the *FUS* (−) + *circ_002136*(−)NC group, the mRNA and protein expression levels of *SOX13* and *SPON2* were further significantly decreased in the *FUS*(−) + *circ_002136*(−) group (Additional file 2: Figure S2B-D). In addition, the effects of dual silencing of *FUS* and *circ_002136* on viability, migration and tube formation of GECs were consistent with the changes in *SOX13* and *SPON2* expression (Additional file 2: Figure S2E-G).

### *Circ_002136*, but not linear *CDK11A*, is up-regulated in GECs, while silencing of *circ_002136* inhibits glioma angiogenesis

**Fig. 2:**
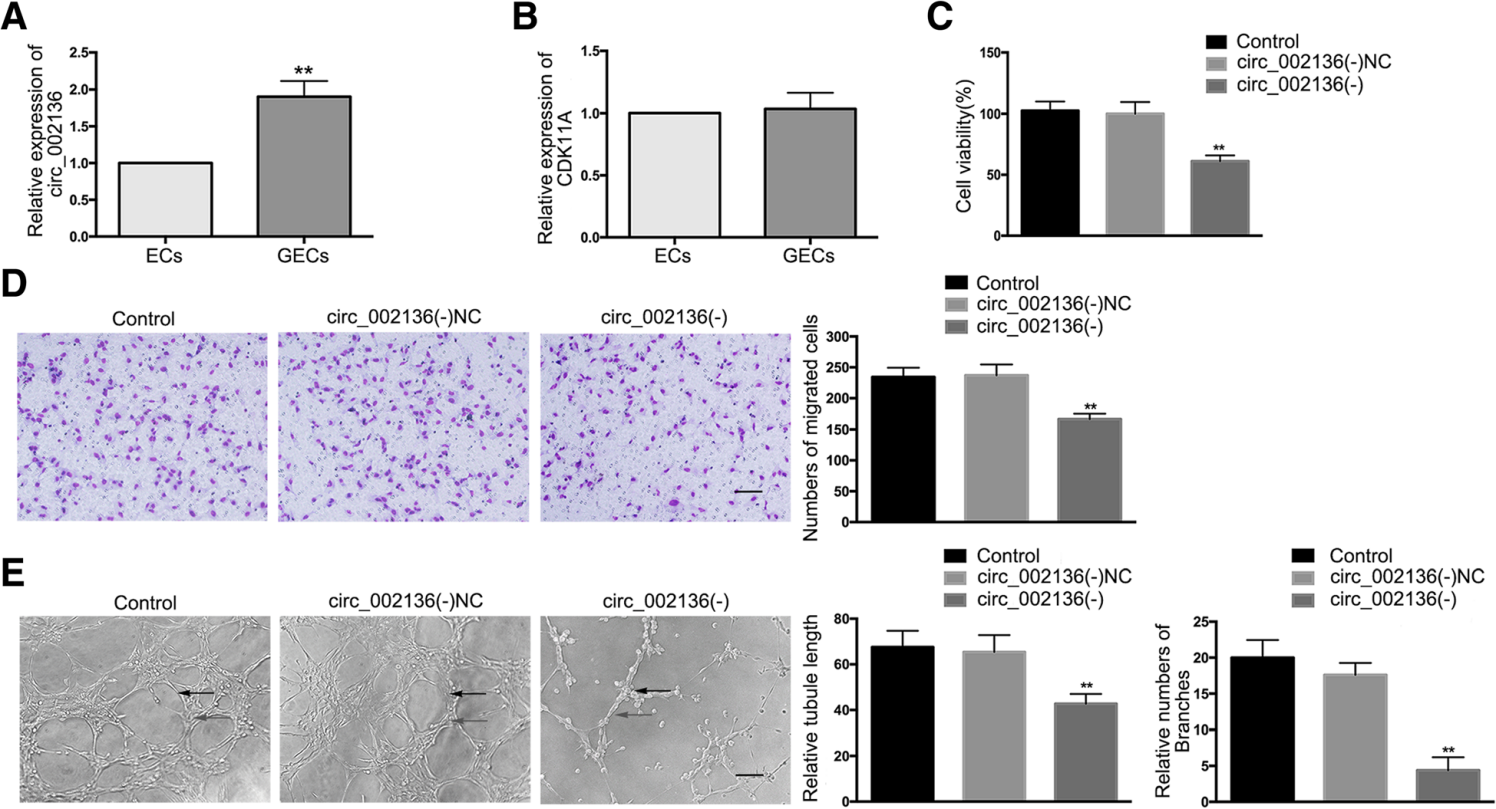
Knockdown of *circ_002136* inhibited GEC angiogenesis in U87 glioma. (**a**) The relative expression of *circ_002136* was detected in ECs and GECs by qRT-PCR. Data are presented as the means ± SD (*n* = 5, each group). ^**^P < 0.01 vs. ECs group. (**b**) The relative expression of *CDK11A* was detected in ECs and GECs by qRT-PCR. Data are presented as the means ± SD (*n* = 5, each group). (**c**) The effects of *circ_002136* knockdown on the viability of GECs were detected by the CCK-8 assay. Values represent the means ± SD (*n* = 5, each group). ^**^P < 0.01 vs. *circ_002136*(−)NC group. (**d**) The effects of *circ_002136* knockdown on the migration of GECs were determined by the transwell assay. Values represent the means ± SD (*n* = 5, each group). ^**^P < 0.01 vs. *circ_002136*(−)NC group. The scale bar represents 100 μm. (E) The effects of *circ_002136* knockdown on the tube formation of GECs were evaluated by the Matrigel tube formation assay. Values represent the means ± SD (n = 5, each group). ^**^P < 0.01 vs. *circ_002136*(−)NC group. The scale represents 100 μm

The expression of *circ_002136* in ECs and GECs was examined. The results showed significantly up-regulated expression of *circ_002136* in the GECs group compared to the ECs group (Fig. 2A). However, the difference in expression of *CDK11A* was not statistically significant between the ECs and GECs groups (Fig. 2B).

RNase R is an RNA exonuclease that degrades linear RNAs with short 3′ tails regardless of their secondary structure, but it does not degrade circular forms. In this study, RNase R was used to confirm the circular form of RNAs. *Circ_002136* was resistant to RNase R treatment, whereas linear *CDK11A* was significantly reduced in ECs and GECs treated with RNase R (Figs. S1B, C). Silencing of *circ_002136* was performed to further investigate the potential effect of *circ_002136* in GECs and transfection efficiency was evaluated by qRT-PCR (Fig. S1D). In addition, the expression of *CDK11A* was detected after silencing of *circ_002136* to confirm that the circular form, rather than the linear form, of *CDK11A* was inhibited. As shown in Fig. S1E, there was no significant difference in *CDK11A* expression between the *circ_002136*(−) and *circ_002136*(−)NC groups. Next, *CDK11A* was silenced to detect whether sh-*CDK11A* influenced *circ_002136* expression. The transfection efficiency of *CDK11A* was verified (Additional file 1: Figure S1F) and there was no significant change in *circ_002136* expression between the sh-*CDK11A* and sh-NC groups (Additional file 1: Figure S1G). Furthermore, cell viability, migration and tube formation abilities were significantly reduced in the *circ_002136*(−) group compared to the *circ_002136*(−)NC group (Fig. 2C–E).

### *MiR-138-5p* is down-regulated in GECs and over-expression of *miR-138-5p* inhibits gliomas angiogenesis

**Fig. 3:**
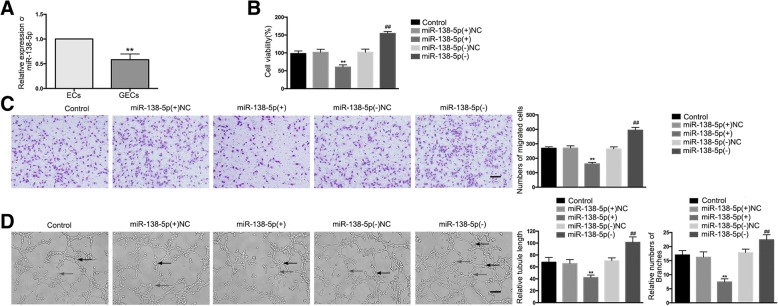
Over-expression of *miR-138-5p* suppressed GEC angiogenesis in U87 glioma. (**a**) The relative expression of *miR-138-5p* in ECs and GECs was detected by qRT-PCR. U6 was used as an internal control. Data are presented as the means ± SD (n = 5, each group). ^**^P < 0.01 vs. ECs group. (**b**) The effects of *miR-138-5p* on the viability of GECs were determined by the CCK-8 assay. Values represent the means ± SD (n = 5, each group). ^**^P < 0.01 vs. *miR-138-5p*(+)NC group; ^##^*P* < 0.01 vs. *miR-138-5p*(−)NC group. (**c**) The effects of *miR-138-5p* on the migration of GECs were determined by the transwell assay. Values represent the means ± SD (n = 5, each group). ^**^P < 0.01 vs. *miR-138-5p*(+)NC group; ^##^*P* < 0.01 vs. *miR-138-5p*(−)NC group. The scale bar represents 100 μm. (**d**) The effects of *miR-138-5p* on tube formation of GECs were evaluated by the Matrigel tube formation assay. Values represent the means ± SD (n = 5, each group). ^**^P < 0.01 vs. *miR-138-5p*(+)NC group; ^##^*P* < 0.01 vs. *miR-138-5p*(−)NC group. The scale bar represents 100 μm

The endogenous expression of *miR-138-5p* in ECs and GECs was detected by qRT-PCR. The expression of *miR-138-5p* was significantly down-regulated in the GECs group compared to the ECs group (Fig. 3A). In this study, over-expression or silencing of *miR-138-5p* was performed to further understand its role in the angiogenesis of GECs, with the transfection efficiency shown in Additional file 1: Figure S1H. The results demonstrated that, compared to the *miR-138-5p*(+)NC group, cell viability, migration, and tube formation abilities were significantly decreased in the *miR-138-5p*(+) group, whereas compared to the *miR-138-5p*(−)NC group, these abilities were significantly increased in the *miR-138-5p*(−) group (Fig. 3B–D).

### *MiR-138-5p* targets *circ_002136* but not *CDK11A*, and reverses *circ_002136*-mediated gliomas angiogenesis

**Fig. 4:**
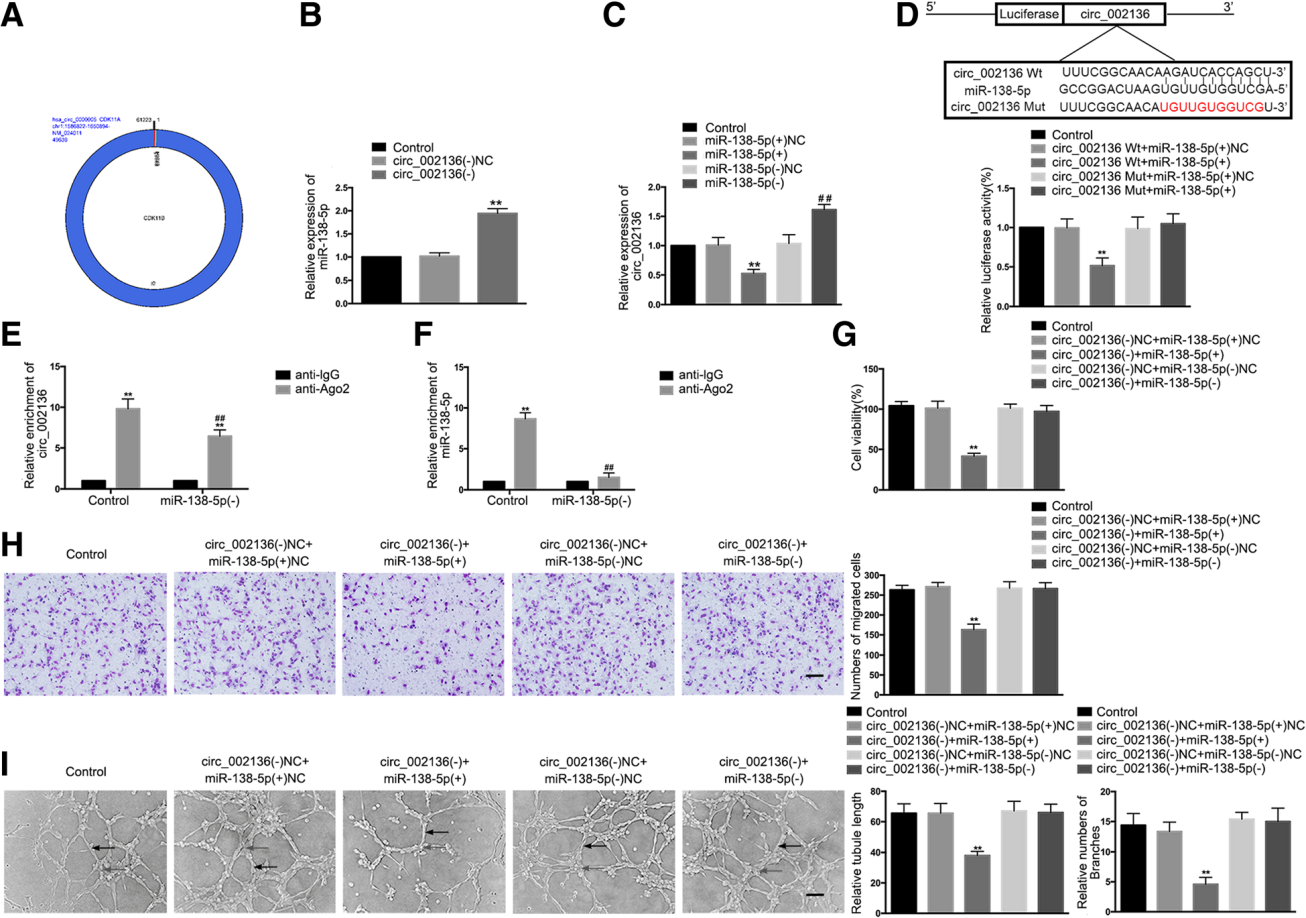
*MiR-138-5p* functionally targeted *circ_002136*. (**a**) A schematic representation of how *circ_002136* arose from the *CDK11A* gene as determined by scanning *CDK11A* genomic DNA and circBase. (**b**) The expression of *miR-138-5p* was measured after knockdown of *circ_002136* by qRT-PCR. Values represent the means ± SD (n = 5, each group). ^**^P < 0.01 vs. *circ_002136*(−)NC group. (**c**) *MiR-138-5p* regulated the expression of *circ_002136* in GECs. Values represent the means ± SD (*n* = 5, each group). ^**^P < 0.01 vs. *miR-138-5p*(+)NC group; ^##^*P* < 0.01 vs. *miR-138-5p*(−)NC group. (**d**) The putative binding site between *circ_002136* and *miR-138-5p* was predicted, and the relative luciferase activity was expressed as firefly/renilla luciferase activity. Values represent the means ± SD (*n* = 5, each group). ^**^P < 0.01 vs. *circ_002136* Wt + *miR-138-5p*(+)NC group. (**e**-**f**) *MiR-138-5p* was identified in the *circ_002136*-RISC complex. The relative expression of *circ_002136* and *miR-138-5p* was measured using qRT-PCR. Data are presented as the means ± SD (*n* = 5, each group). ^**^P < 0.01 vs. anti-IgG group, ^##^*P* < 0.01 vs. anti-Ago2 in control group. (**g**) The co-effects of *circ_002136* and *miR-138-5p* on the viability of GECs were evaluated by the CCK-8 assay. Data are presented as the means ± SD (n = 5, each group). ^**^P < 0.01 vs. *circ_002136*(−)NC + *miR-138-5p*(+)NC group. (**h**) The co-effects of *circ_002136* and *miR-138-5p* on the migration of GECs were evaluated by the transwell assay. Data are presented as the means ± SD (*n* = 5, each group). ^**^P < 0.01 vs. *circ_002136*(−)NC + *miR-138-5p*(+)NC group. The scale bar represents 100 μm. (I) The co-effects of *circ_002136* and *miR-138-5p* on the tube formation of GECs were evaluated by the Matrigel tube formation assay. Data are presented as the means ± SD (*n* = 5, each group). ^**^P < 0.01 vs. *circ_002136*(−)NC + *miR-138-5p*(+)NC group. The scale bar represents 100 μm

The sequence of *circ_002136* (chr1: 1586822–1,650,894) was characterized by alignment with the *CDK11A* genomic DNA sequence (Fig. 4A). The expression of *miR-138-5p* was significantly enhanced in the *circ_002136*(−) group compared to the *circ_002136*(−)NC group (Fig. 4B). Similarly, it was found that over-expression or silencing of *miR-138-5p* resulted in significantly decreased or increased expression of *circ_002136* in GECs (Fig. 4C). However, over-expression or silencing of *miR-138-5p* did not affect the expression of linear *CDK11A* (Additional file 1: Figure S1I).

Using the bioinformatics software Starbase v2.0 (http://starbase.sysu.edu.cn/), it was predicted that *circ_002136* might possess a binding site for *miR-138-5p*. This prediction was verified by a dual-luciferase reporter assay system. The results showed that compared to the *circ_002136*Wt + *miR-138-5p*(+)NC group, the relative luciferase activity of the *circ_002136*Wt + *miR-138-5p*(+) group was significantly suppressed. Nevertheless, there were no significant differences in relative luciferase activity between the *circ_002136*Mut + *miR-138-5p*(+) and *circ_002136*Mut + *miR-138-5p*(+)NC groups (Fig. 4D). Furthermore, RNA-IP results showed that compared to the anti-IgG group, the relative abundance of *circ_002136* and *miR-138-5p* in the anti-Ago2 group significantly increased, whereas in the *miR-138-5p* (−) group, the relative abundance of *circ_002136* and *miR-138-5p* immunoprecipitated with Ago2 was lower than that in the control group. This suggests that *circ_002136* and *miR-138-5p* were co-enriched in the expected RISC (Fig. 4E, F).

To further investigate whether *circ_002136* regulates glioma angiogenesis by modulating *miR-138-5p*, GECs with stable knockdown of *circ_002136* were transiently transfected with *miR-138-5p* agomir or antagomir. Rescue experiments showed that cell viability, migration, and tube formation abilities were significantly reduced in the *circ_002136*(−) + *miR-138-5p*(+) group compared to the *circ_002136*(−)NC + *miR-138-5p*(+)NC group, whereas there was no significant difference between the *circ_002136*(−) + *miR-138-5p*(−) and *circ_002136*(−)NC + *miR-138-5p*(−)NC groups (Fig. 4G–I).

### *SOX13* is a target gene of *miR-138-5p* and is involved in *circ_002136* and *miR-138-5p*-mediated gliomas angiogenesis

**Fig. 5:**
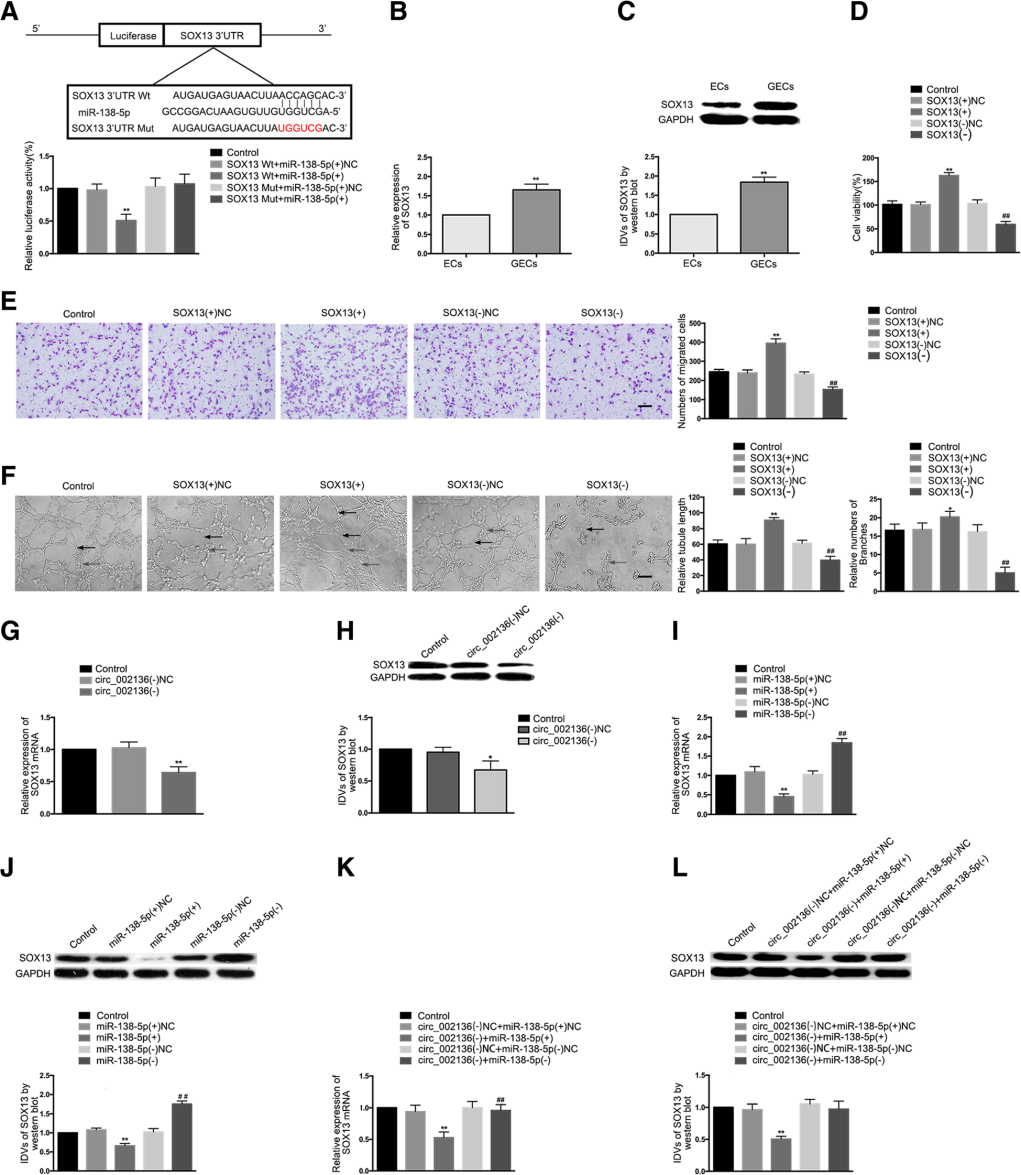
*SOX13*, a target of *miR-138-5p*, regulated the GEC angiogenesis in U87 glioma. (**a**) A putative binding site between *SOX13* and *miR-138-5p* was predicted and the relative luciferase activity was expressed as the firefly/renilla luciferase activity. Values represent the means ± SD (*n* = 5, each group). ^**^P < 0.01 vs. *SOX13* Wt + *miR-138-5p*(+)NC group. (**b**-**c**) The mRNA and protein expression levels of *SOX13* were measured in ECs and GECs by qRT-PCR and western blot. Values represent the means ± SD (*n* = 5, each group). ^**^P < 0.01 vs. ECs group. (**d**) *SOX13* regulated the viability of GECs. Values represent the means ± SD (n = 5, each group). ^**^P < 0.01 vs. *SOX13*(+)NC group; ^##^*P* < 0.01 vs. *SOX13*(−)NC group. (**e**) *SOX13* regulated the migration of GECs. Values represent the means ± SD (n = 5, each group). ^**^P < 0.01 vs. *SOX13*(+)NC group; ^##^*P* < 0.01 vs. *SOX13*(−)NC group. The scale bar represents 100 μm. (**f**) *SOX13* regulated the tube formation of GECs. Values represent the means ± SD (n = 5, each group). ^*^P < 0.05, ^**^P < 0.01 vs. *SOX13*(+)NC group; ^##^*P* < 0.01 vs. *SOX13*(−)NC group. The scale bar represents 100 μm. (**g**-**h**) The mRNA and protein expression levels of *SOX13* were inhibited after knockdown of *circ_002136*. Values represent the means ± SD (*n* = 3, each group). ^*^P < 0.05, ^**^P < 0.01 vs. *circ_002136*(−)NC group. (**i**-**j**) The mRNA and protein expression levels of *SOX13* were regulated by *miR-138-5p*. Values represent the means ± SD (*n* = 3, each group). ^**^P < 0.01 vs. *miR-138-5p*(+)NC group. ^##^*P* < 0.01 vs. *miR-138-5p*(−)NC group. (K-L) The mRNA and protein expression levels of *SOX13* were co-regulated by both *circ_002136* and *miR-138-5p*. Values represent the means ± SD (n = 3, each group). ^**^P < 0.01 vs. *circ_002136*(−)NC + *miR-138-5p*(+)NC group

Using the bioinformatics software microRNA.org-target program (http://www.microrna.org/microrna/home.do), it was predicted that a putative binding site for *miR-138-5p* exists in the 3’-UTR of *SOX13* mRNA, which was a complementary sequence of the *miR-138-5p* seed region. The results of the dual-luciferase reporter assay showed that compared to the *SOX13*–3’-UTR-Wt + *miR-138-5p*(+)NC group, the relative luciferase activity in the *SOX13*–3’-UTR-Wt + *miR-138-5p*(+) group was significantly reduced, while compared to the *SOX13*–3’-UTR-Mut + *miR-138-5p*(+)NC group, the relative luciferase activity in the *SOX13*–3’-UTR-Mut + *miR-138-5p*(+) group showed no significant change (Fig. 5A).

The mRNA and protein expression levels of *SOX13* in the GECs group were significantly up-regulated compared to the ECs group (Fig. 5B, C). In this study, both over-expression and silencing of *SOX13* were performed in GECs to further explore the effect of *SOX13* on the angiogenesis of GECs. The stable transfection efficiency of *SOX13* was also verified (Additional file 1: Figure S1J). As shown in Fig. 5D-F, compared to the *SOX13*(+)NC group, the cell viability, migration, and tube formation abilities of the *SOX13*(+) group were significantly improved, while compared to the *SOX13*(−)NC group, these abilities in the *SOX13*(−) group were significantly reduced.

This study further investigated whether *SOX13* is involved in the regulatory network of *circ_002136* and *miR-138-5p* in glioma angiogenesis. The results showed that the mRNA and protein expression levels of *SOX13* were significantly reduced in the *circ_002136*(−) group compared to the *circ_002136*(−)NC group (Fig. 5G, H). Compared to the *miR-138-5p*(+)NC group, however, the mRNA and protein expression levels of *SOX13* in the *miR-138-5p*(+) group were significantly reduced. Compared to the *miR-138-5p*(−)NC group, the mRNA and protein expression levels of *SOX13* in the *miR-138-5p*(−) group were significantly increased (Fig. 5I, J). Furthermore, the rescue experiments showed that the mRNA and protein expression levels of *SOX13* were significantly reduced in the *circ_002136*(−) + *miR-138-5p*(+) group compared to the *circ_002136*(−)NC + *miR-138-5p*(+)NC group, but showed no significant differences in the *circ_002136*(−) + *miR-138-5p*(−) group compared to the *circ_002136*(−)NC + *miR-138-5p*(−)NC group (Fig. 5K, L).

### *SOX13* binds to the *SPON2* promoter region to promote expression of *SPON2*

**Fig. 6:**
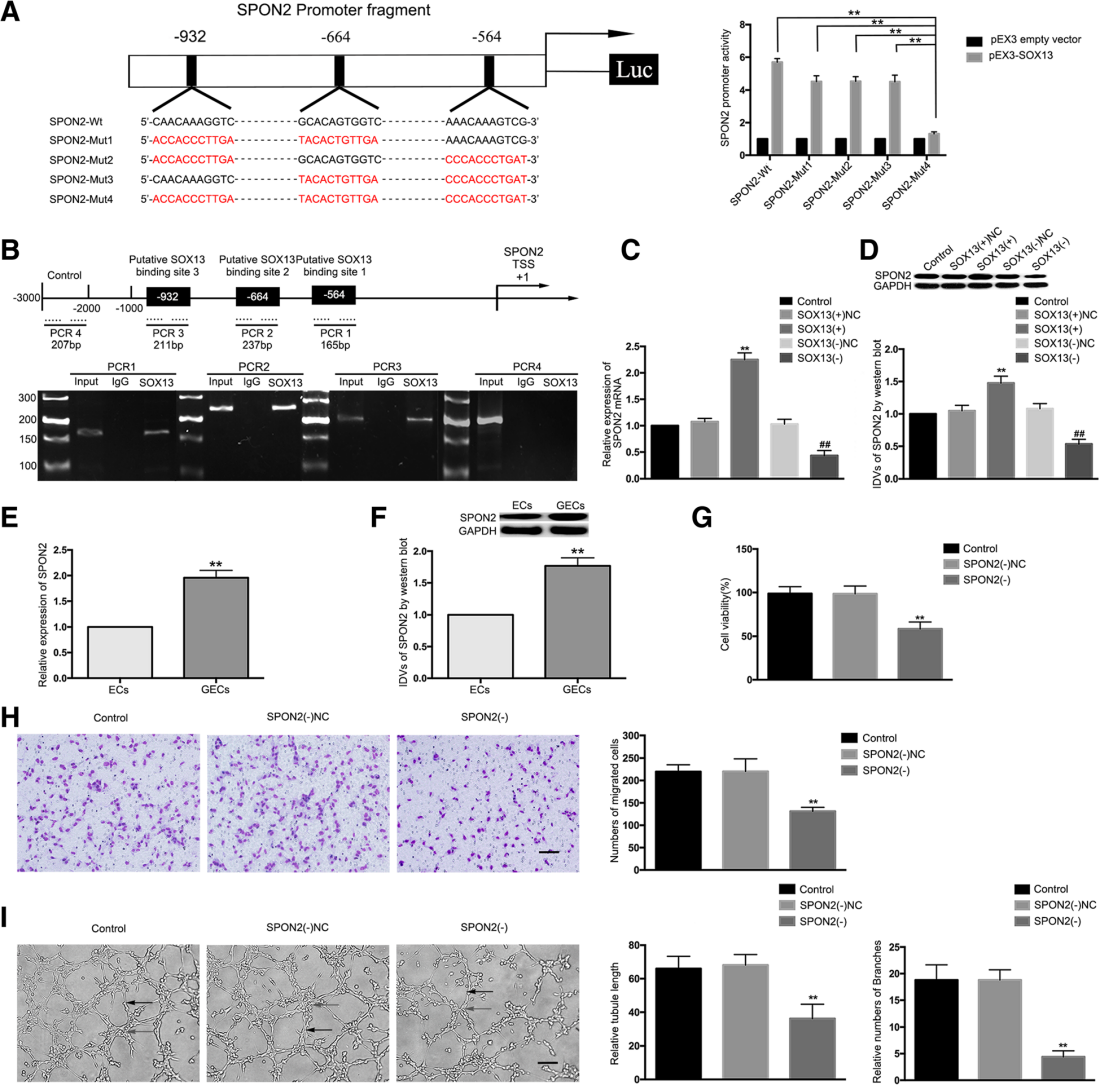
*SPON2* was a target of *SOX13*; *SPON2* knockdown suppressed the GEC angiogenesis in U87 glioma. (**a**) A schematic depiction of the predicted binding sites for *SOX13* on the promoter fragment of *SPON2* and the site mutagenesis design for the reporter assay. Promoter activities were measured to determine responsive *SOX13*-binding sites in the promoter of *SPON2* using luciferase reporter assays. Data are presented as the means ± SD (n = 3, each group). ^**^P < 0.01 vs. *SPON2*-Mut4 group. (**b**) A schematic representation of the *SPON2* promoter region 3000 bp upstream or 200 bp downstream of the TSS, designated as + 1. ChIP PCR products for the putative *SOX13* binding sites and an upstream region not expected to associate with *SOX13* are depicted with bold lines. (**c**-**d**) The qRT-PCR and western blot analysis of the *SOX13* regulation of *SPON2* mRNA and protein expression levels. Values represent the means ± SD (n = 3, each group). ^**^P < 0.01 vs. *SOX13*(+)NC group; ^##^*P* < 0.01 vs. *SOX13*(−)NC group. (**e**-**f**) The mRNA and protein expression levels of *SPON2* in ECs and GECs were evaluated by qRT-PCR and western blot. Data are presented as the means ± SD (n = 5, each group). ^**^P < 0.01 vs. ECs group. (**g**) The effects of *SPON2* knockdown on the viability of GECs were detected by the CCK-8 assay. Values represent the means ± SD (n = 5, each group). ^**^P < 0.01 vs. *SPON2*(−)NC group. (**h**) The effects of *SPON2* knockdown on the migration of GECs were determined by the transwell assay. Values represent the means ± SD (n = 5, each group). ^**^P < 0.01 vs. *SPON2*(−)NC group. The scale bar represents 100 μm. (**i**) The effects of *SPON2* knockdown on the tube formation of GECs were evaluated by the Matrigel tube formation assay. Values represent the means ± SD (n = 5, each group). ^**^P < 0.01 vs. *SPON2*(−)NC group. The scale bar represents 100 μm

A search of the bioinformatics software JASPAR (http://jaspar.genereg.net) database predicted three putative *SOX13*-binding sites at loci 564, 664, and 932 in the 1000-bp region upstream and 200-bp region downstream of the *SPON2* transcription start site (TSS). To determine the regions responsible for the *SOX13* activated promoter of *SPON2* in GECs, a series of mutation constructs were generated and luciferase reporter assays were performed. Wild-type and mutation constructs are indicated in Fig. 6A. As shown in Fig. 6A, *SPON2* promoter activities were up-regulated after co-transfecting with pEX3-*SOX13*. Mutation of all the putative *SOX13* binding sites induced a significant change in *SPON2* promoter activity, but mutation of any two did not. These results established that *SOX13* stimulated the promoter activity of *SPON2* and indicated that the *SPON2* responsive elements, which were necessary for high promoter activity, were likely to reside within the 564, 664, and 932 site regions. To further determine whether *SOX13* was directly associated with the *SPON2* promoter, ChIP assays were performed. ChIP assays revealed that *SOX13* binds to the *SPON2* promoter region, with no relationship observed in the control region (Fig. 6B). Subsequently, the mRNA and protein expression levels of *SPON2* were examined in the GECs with stable over-expression or silencing of *SOX13*. Compared to the *SOX13*(+)NC group, the mRNA and protein expression levels of *SPON2* in the *SOX13*(+) group were significantly increased, while the mRNA and protein expression levels of *SPON2* in the *SOX13*(−) group were significantly decreased compared to those in the *SOX13*(−)NC group (Fig. 6C, D).

In this study, the expression of *SPON2* in ECs and GECs was further examined. The results showed that the mRNA and protein expression levels of *SPON2* in the GECs group were significantly up-regulated compared to the ECs group (Fig. 6E, F). *SPON2* was then silenced to further investigate its function in GECs. The stable transfection efficiency of SPON2 is shown in Additional file 1: Figure S1K. Cell viability, migration, and tube formation in the *SPON2*(−) group were significantly reduced compared to the *SPON2*(−)NC group (Fig. 6G–I).

### *MiR-138-5p* suppresses *SOX13*-mediated angiogenesis and the expression of *SPON2*; *SOX13* promotes *FUS* transcription to form a feedback loop regulating glioma angiogenesis

**Fig. 7:**
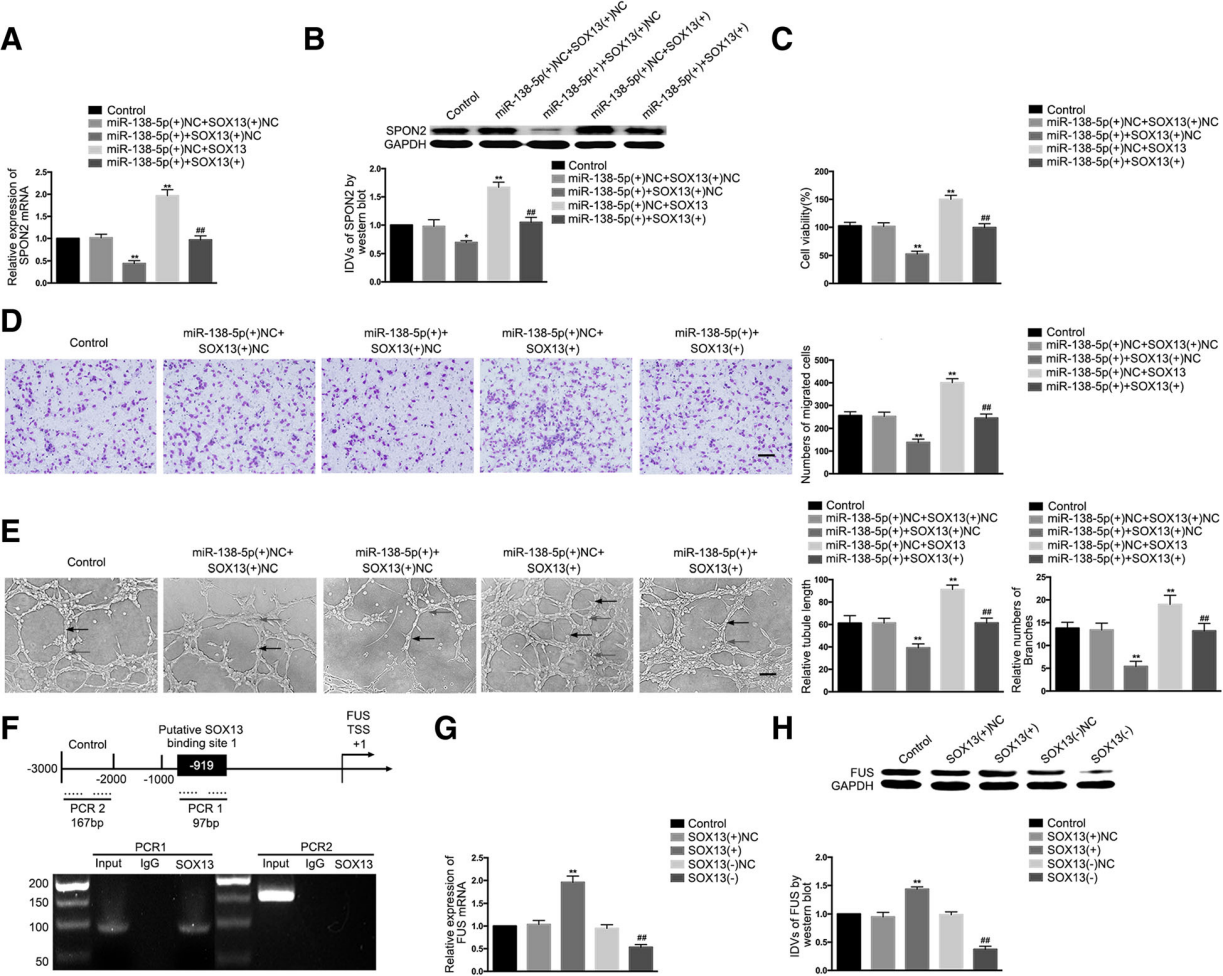
Co-effects of *miR-138-5p* and *SOX13*; *SOX13* promoted transcription of *FUS* forming a feedback loop. (**a**-**b**) The co-effects of *miR-138-5p* and *SOX13* on the expression levels of *SPON2* were evaluated by qRT-PCR and western blot. Data are presented as the means ± SD (n = 3, each group). ^*^P < 0.05, ^**^P < 0.01 vs. *miR-138-5p*(+)NC + *SOX13*(+)NC group. ^##^*P* < 0.01 vs. *miR-138-5p*(+) + *SOX13*(+)NC group. (**c**) The co-effects of *miR-138-5p* and *SOX13* on the viability of GECs were evaluated by the CCK8 assay. Data are presented as the means ± SD (n = 5, each group). ^**^P < 0.01 vs. *miR-138-5p*(+)NC + *SOX13*(+)NC group; ^##^*P* < 0.01 vs. *miR-138-5p*(+) + *SOX13*(+)NC group. (**d**) The co-effects of *miR-138-5p* and *SOX13* on the migration of GECs were evaluated by the transwell assay. Data are presented as the means ± SD (n = 5, each group). ^**^P < 0.01 vs. *miR-138-5p*(+)NC + *SOX13*(+)NC group; ^##^*P* < 0.01 vs. *miR-138-5p*(+) + *SOX13*(+)NC group. The scale bar represents 100 μm. (**e**) The co-effects of *miR-138-5p* and *SOX13* on the tube formation of GECs were evaluated by the Matrigel tube formation assay. Data are presented as the means ± SD (n = 5, each group). ^*^P < 0.05, ^**^P < 0.01 vs. *miR-138-5p*(+)NC + *SOX13*(+)NC group; ^##^*P* < 0.01 vs. *miR-138-5p*(+) + *SOX13*(+)NC group. The scale bar represents 100 μm. (**f**) Schematic representation of the human *FUS* promoter region 3000 bp upstream or 200 bp downstream of the TSS, designated as + 1. ChIP PCR products for the putative *SOX13* binding site and an upstream region not expected to associate with *SOX13* are depicted with bold lines. (**g**-**h**) The qRT-PCR and western blot analysis of *SOX13* regulation of the expression levels of *FUS*. Values are presented as the means ± SD (n = 3, each group). ^**^P < 0.01 vs. *SOX13*(+)NC group; ^##^*P* < 0.01 vs. *SOX13*(−)NC group

*MiR-138-5p* was overexpressed in GECs stably transfected with the *SOX13* over-expression vector, and the effect of double over-expression of *miR-138-5p* and *SOX13* on mRNA and protein expression of *SPON2* was examined. Compared to the *miR-138-5p*(+)NC + *SOX13*(+)NC group, the mRNA and protein expression levels of *SPON2* were significantly decreased in the *miR-138-5p*(+) + *SOX13*(+)NC group and significantly increased in the *miR-138-5p*(+)NC + *SOX13*(+) group, whereas *SPON2* expression in the *miR-138-5p*(+) + *SOX13*(+) group was significantly increased compared to the *miR-138-5p*(+) + *SOX13*(+)NC group (Fig. 7A, B). In addition, the changes in viability, migration, and tube formation were consistent with the changes in *SPON2* expression (Fig. 7C–E).

One putative *SOX13* binding site was predicted at loci 919 in the 1000-bp region upstream and 200-bp region downstream of the *FUS* TSS using the JASPAR database. ChIP assays revealed that *SOX13* and *FUS* bound at this putative binding site, but no relationship was observed in the control region (Fig. 7F). Similarly, the mRNA and protein expression levels of *FUS* were examined in GECs with stable over-expression or silencing of *SOX13.* Compared to the *SOX13*(+)NC group, the mRNA and protein expression levels of *FUS* in the *SOX13*(+) group were significantly increased, whereas compared to the *SOX13*(−)NC group, the mRNA and protein expression levels of *FUS* in the *SOX13*(−) group were significantly decreased (Fig. 7G, H).

### *FUS* or *circ_002136* knockdown and *miR-138-5p* over-expression significantly suppress glioma angiogenesis in vivo both individually and combined

**Fig. 8:**
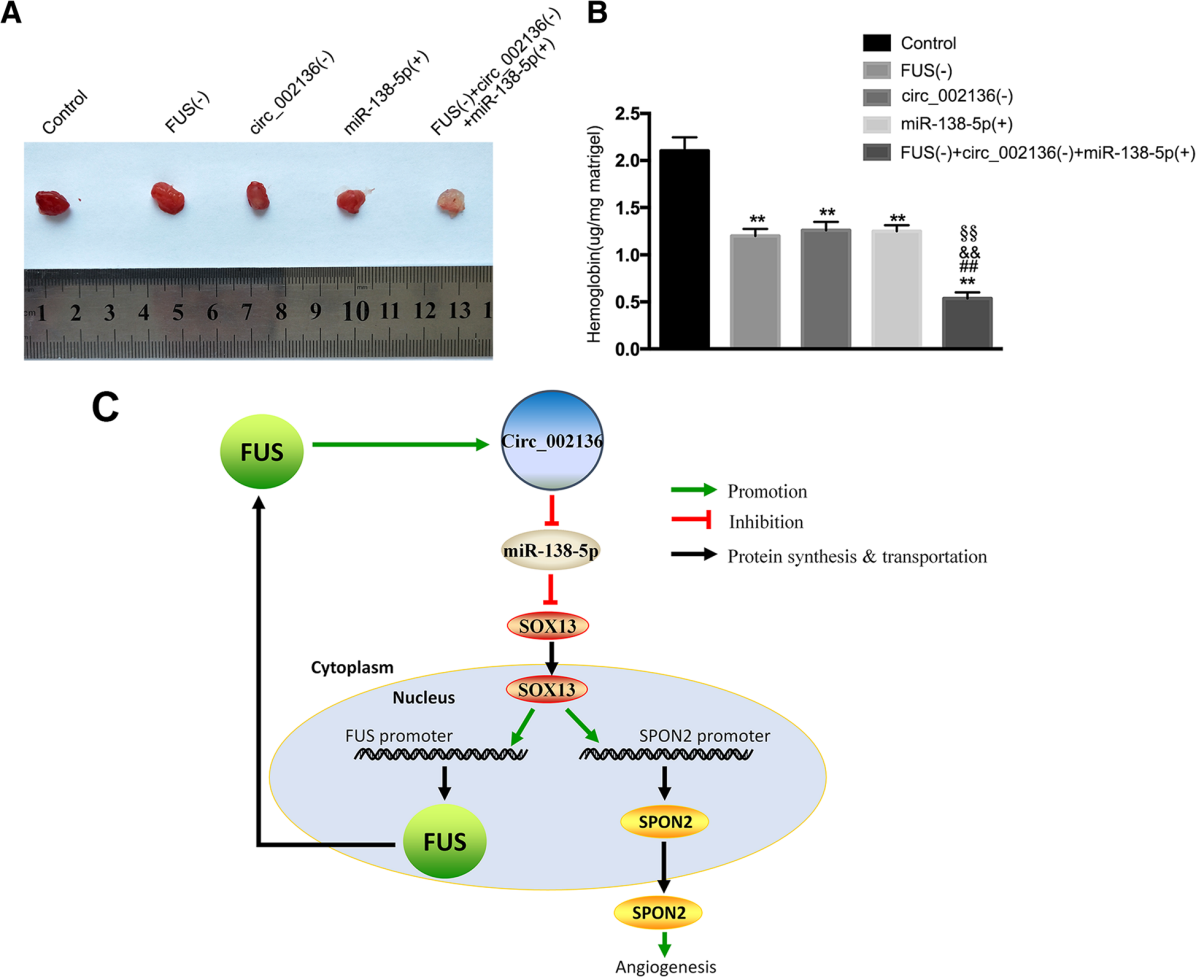
*FUS* and *circ_002136* knockdown combined with *miR-138-5p* over-expression suppressed angiogenesis in vivo (**a**) The co-effect of *FUS*, *circ_002136*, and *miR-138-5p* on angiogenesis in vivo was evaluated by the Matrigel plug assay. (**a**) The amount of hemoglobin was measured after the combined treatment of *FUS*, *circ_002136*, and *miR-138-5p*. Data are presented as the means ± SD (n = 3, each group). ^**^P < 0.01 vs. control group; ^##^*P* < 0.01 vs. the *FUS* (−) group; ^&&^*P* < 0.01 vs. the *circ_002136* (−) group. ^§§^*P* < 0.01 vs. the *miR-138-5p* (+) group. (**c**) A schematic representation of the *FUS/circ_002136/miR-138-5p/SOX13/SPON2* axis involvement in the mechanism mediating the GEC angiogenesis

A matrigel plug assay was applied to further verify the effects of *FUS* or *circ_002136* knockdown and *miR-138-5p* over-expression in vivo. Co-transfection was conducted prior to the assessment of angiogenesis in vivo. The results showed that the amount of hemoglobin in the *FUS*(−), *circ_002136*(−), and *miR-138-5p*(+) groups was significantly decreased, compared to the control group. The amount of hemoglobin was lowest in the *FUS*(−) + *circ_002136*(−) + *miR-138-5p*(+) group (Fig. 8A, B). These findings suggested that silencing of *FUS* and *circ_002136,* combined with *miR-138-5p* over-expression, suppressed glioma angiogenesis in vivo. A schematic representation of the mechanism by which the *FUS*/*circ_002136*/*miR-138-5p*/*SOX13*/*SPON2* axis affects the angiogenesis of GECs is presented in Fig. 8C.

## Discussion

This study demonstrated for the first time that *FUS*, *circ_002136*, *SOX13* and *SPON2* were highly expressed, while *miR-138-5p* was under-expressed in GECs. *FUS* bound to *circ_002136*, while *circ_002136* acted as a molecular sponge for *miR-138-5p*, which had a negative regulatory effect on *SOX13* and regulated glioma angiogenesis. *SOX13* promoted the expression of *SPON2* and increased the angiogenic capacity of GECs. *SOX13* bound to the *FUS* promoter region to up-regulate the expression of *FUS*, forming a positive feedback loop that promoted glioma angiogenesis.

This study found that *FUS* was highly expressed in GECs and that *FUS* silencing inhibited the viability, migration, and tube formation of GECs. It has been reported that *FUS* plays an important role in regulating various biological processes. For example, *FUS* and DNA damage-inducible transcript 3 are able to form a fusion oncogene *FUS*-CHOP, which increases the invasive capability of human mucus-like and round cell liposarcoma cells [21]. *FUS* protein is also a pathological hallmark of neurodegenerative diseases such as amyotrophic lateral sclerosis and frontotemporal lobe degeneration [22, 23]. This study further found that *circ_002136* was highly expressed in GECs and that *circ_002136* silencing was reduced in GEC angiogenesis, suggesting that *circ_002136* functioned as an oncogene in GECs.

The regulation of glioma development by circRNAs has become a major research focus. *Circ_0001649* regulates glioma cell growth, colony formation, and apoptosis [24]. *Circ-FBXW7* encodes FBXW7-185aa, which can shorten the half-life of c-Myc and slow the proliferation of glioma cells [25]. CircRNAs also play an important role in regulating EC function. *Circ_0003575* is up-regulated in oxLDL-induced human umbilical vein endothelial cells (HUVECs) and promotes HUVEC proliferation and angiogenic capacity [26]. *CircHECTD1* accelerates silica-induced transformation from pulmonic ECs to mesenchymal cells [27]. As previously reported, *CDK11A* is abnormally expressed in penile squamous cell carcinoma [28] and hepatocellular carcinoma [29]. In this study, we observed that the expression of linear *CDK11A* was not up-regulated in GECs and that the degradation of *CDK11A* by RNase R did not affect the expression of *circ_002136*, suggesting that *circ_002136* and linear *CDK11A* are two mutually independent RNAs that might perform different functions, a finding consistent with the function of *circ-SHKBP1* [30].

The present study revealed that *FUS* contained a binding site for *circ_002136*, which was proven through RNA-IP and RNA pull-down assays. It was further confirmed that *FUS* silencing significantly reduced the expression of *circ_002136*, while the expression of *CDK11A* was not affected. Previous studies have shown that RBPs can interact with circRNAs to perform diverse biological functions. In doxorubicin-induced heart failure, the RBP *Qki5* interacts with circRNAs *Ttn*, *Fhod3*, and *Strn3* to improve cardiac function [31]. In lung cancer cells, the RBP *TNRC6A* binds to and regulates the formation of *circ_0006916* accelerating lung cancer cell growth [32].

Our findings demonstrated that *miR-138-5p* was down-regulated in GECs and that *miR-138-5p* over-expression significantly reduced the angiogenic capacity of GECs, and vice versa, suggesting that *miR-138-5p* acted as a tumor suppressor in GECs. These findings followed those of other research groups. *MiR-138-5p* inhibits proliferation and enhances radiation-induced DNA damage and autophagy in nasopharyngeal carcinoma [33]. *MiR-138-5p* contributed to the TNF-α-induced insulin resistance through inducing autophagy in HepG2 cells by regulating SIRT1 [34]. Our study also verified that a binding site existed between *circ_002136* and *miR-138-5p*, indicating that *circ_002136* might act as a *miR-138-5p* sponge to modulate its functions in GECs. Further studies manifested that knockdown of *circ_002136* significantly up-regulated the expression of *miR-138-5p*. *MiR-138-5p* silencing increased the expression of *circ_002136* and vice versa. These findings, as well as those from the RNA-IP assays, suggested reciprocal repression between *circ_002136* and *miR-138-5p*, which might operate in a RISC manner. Furthermore, the co-effect of *circ_002136* and *miR-138-5p* on the angiogenesis of GECs was verified. The results showed that *miR-138-5p* reversed *circ_002136* knockdown-mediated inhibition of viability, migration, and tube formation of GECs. Accumulated evidence confirms that circRNAs serve as miRNA sponges by targeting miRNAs. For example, circRNA *ZNF609* functions as a competitive endogenous RNA to regulate *FOXP4* expression by sponging *miR-138-5p* in renal carcinoma [35].CircRNA *MYLK* binds to *miR-29a* and promotes epithelial-mesenchymal transition and xenograft growth, angiogenesis, and metastasis of bladder cancer [36].

This study confirmed high expression of *SOX13* in GECs. *SOX13*-silencing attenuated the angiogenic capacity of GECs and vice versa, indicating that *SOX13* might act as an oncogene in GECs. *SOX13* acts as an effective regulator of embryonic development, stem cell maintenance, tissue homeostasis, and multiple cancer development [37]. Previous studies have confirmed that *SOX13* is up-regulated in tumors such as renal clear cell carcinoma [38] and colorectal cancer [39]. *SOX13* regulates T lymphocyte differentiation by promoting the development of γδ T cells and inhibiting the differentiation of αβ T cells [40].

One of the most common modes of miRNAs action is the inhibition of gene expression at the transcriptional and post-transcriptional levels by binding to the 3′-UTR of the target mRNA [41]. Our results revealed that *SOX13* was a target gene for *miR-138-5p* and that *miR-138-5p* binds to the ACCAGC sequence in the 3’-UTR of *SOX13*. In GECs, *circ_002136* silencing significantly reduced the expression of *SOX13*, while over-expression and silencing of *miR-138-5p* significantly reduced or increased, respectively, the expression of *SOX13*. Further studies revealed that the reduction in *SOX13* expression mediated by *circ_002136* knockdown was reversed by *miR-138-5p* silencing. *MiR-138-5p* performs many biological functions by regulating target genes. For example, in non-small cell lung cancer, *miR-138-5p* reduces the expression of *GPR124* and suppresses Gefitinib resistance [42].

Our present study demonstrated that *SPON2* performed oncogene functions in GECs as a result of *SPON2* up-regulating angiogenesis. This was confirmed by the inhibition of angiogenesis in *SPON2*-knockdown GECs. *SPON2* is an innate immune modulator of host cells that recruits inflammatory cells and regulates neuronal development. Previous studies have shown that *SPON2* plays an important role in Egr-1-mediated inhibition of proliferation, migration, and tube formation of vascular ECs in colorectal cancer [43]. In addition, luciferase reporter assays and ChIP assays certified that *SOX13* was directly associated with and activated the *SPON2* promoter, thereby up-regulating *SPON2* expression. Further findings demonstrated that over-expression of *SOX13* significantly increased the expression of *SPON2* and that *SOX13* promoted the angiogenesis of GECs by transcriptionally up-regulating *SPON2*. Moreover, our data indicated that *miR-138-5p* down-regulated the expression of *SPON2* and inhibited the viability, migration, and tube formation of GECs by negatively regulating *SOX13*. Based on the above results, knockdown of *circ_002136* negatively regulated the expression of *SOX13* by targeting *miR-138-5p* and further down-regulated *SPON2* expression to inhibit the glioma angiogenesis.

Based on our prediction, ChIP assays were performed to confirm that *SOX13* bound to the *FUS* promoter and promoted *FUS* transcription. Over-expression of *SOX13* up-regulated *FUS* expression, whereas inhibition of *SOX13* diminished *FUS* expression, validating our hypothesis that a positive feedback loop was formed between *FUS*/*circ_002136*/*miR-138-5p*/*SOX13* to regulate the angiogenesis of GECs. Similar positive feedback loops have been reported in previous studies. In the *TDP43/SNHG12/miR-195/SOX5* pathway of glioma cells, *SOX5* promotes the transcription of *SNHG12* and forms a positive feedback loop to regulate the biological behavior of glioma cells [44]. *In the GAS5/miR-196a-5p/FOXO1/PID1 (MIIP)* pathway of glioma stem cells, *FOXO1* promotes *GAS5* transcription and forms a positive feedback loop that regulates the biological behavior of glioma stem cells [45]. Regulation of the positive feedback loop and its effect on the function of glioma cells and angiogenesis is now an important research focal point in biomedical science.

## Conclusions

In summary, the present study revealed that knockdown of *FUS* and *circ_002136* inhibited the viability, migration, and tube formation of GECs. *MiR-138-5p* exerted angiogenesis-suppressive function by decreasing *SOX13* expression. The results of this study for the first time provided insights into the multi-level molecular regulatory networks formed by *FUS* (RBP), *circ_002136* (circRNAs), *miR-138-5p* (miRNAs), *SOX13* (transcription factor), and *SPON2* (target gene). In conclusion, our findings provide a better understanding of RBP-circRNA-miRNA- transcription factor feedback loop function in glioma angiogenesis. *FUS/circ_002136/miR-138-5p/SOX13/SPON2* may aid in slowing the gliomagenesis in patients and provide an alternative strategy for glioma treatment.

## Additional files

Additional file 1: **Figure S1.** Correlation between *circ_002136*, *miR-138-5p* and linear *CDK11A* and evaluation of the transfection efficiency. (A) The transfection efficiency of *FUS*. Data are presented as the means ± SD (*n* = 3, each group). ^**^P < 0.01 vs. *FUS* (−) NC group. (B) The expression of *circ_002136* with RNase R treatment. Data are presented as the means ± SD (*n* = 5, each group). ^**^P < 0.01 vs. control group in ECs; ^##^*P* < 0.01 vs. RNase R group in ECs. (C) The expression of *CDK11A* with RNase R treatment. Data are presented as the means ± SD (*n* = 5, each group). ^**^P < 0.01 vs. control group in ECs; ^##^*P* < 0.01 vs. control group in ECs. (D) The transfection efficiency of *circ_002136* knockdown. Data are presented as the means ± SD (n = 5, each group). ^**^P < 0.01 vs. *circ_002136* (−) NC group. (E) The expression of *CDK11A* after *circ_002136* knockdown. Data are presented as the means ± SD (n = 5, each group). (F) The transfection efficiency of *CDK11A* knockdown. Data are presented as the means ± SD (n = 5, each group). ^**^P < 0.01 vs. sh-NC group. (G) The expression of *circ_002136* after *CDK11A* knockdown. Data are presented as the means ± SD (n = 5, each group). (H) The transfection efficiency of *miR-138-5p* agomir or antagomir. Data are presented as the means ± SD (n = 5, each group). ^**^P < 0.01 vs. *miR-138-5p* (+) NC group, ^##^*P* < 0.01 vs. *miR-138-5p* (−) NC group. (I) The expression of *CDK11A* after *miR-138-5p* over-expression or silencing. Data are presented as the means ± SD (n = 5, each group). (J) The transfection efficiency of *SOX13*. Data are presented as the means ± SD (n = 3, each group). ^**^P < 0.01 vs. *SOX13* (+) NC group, ^##^*P* < 0.01 vs. *SOX13* (−) NC group. (K) The transfection efficiency of *SPON2*. Data are presented as the means ± SD (n = 3, each group). ^**^P < 0.01 vs. *SPON2* (−) NC group. (TIF 471 kb)

Additional file 2: **Figure S2.** Co-effects of *FUS* and *circ_002136* on the expression of downstream molecules and angiogenesis. (A) The expression of *miR-138-5p* was co-regulated by both *FUS* and *circ_002136*. Values represent the means ± SD (n = 5, each group). ^**^P < 0.01 vs. *FUS*(−)NC + *circ_002136*(−)NC group. ^##^*P* < 0.01 vs. *FUS* (−) + *circ_002136*(−)NC group. (B-D) The co-effects of *FUS* and *circ_002136* on the mRNA and protein expression levels of *SOX13* and *SPON2* in GECs were evaluated by qRT-PCR and western blot. Data are presented as the means ± SD (n = 3, each group). ^**^P < 0.01 vs. *FUS*(−)NC + *circ_002136*(−)NC group. ^##^*P* < 0.01 vs. *FUS*(−) + *circ_002136*(−)NC group. (E) The co-effects of *FUS* and *circ_002136* on the viability of GECs were evaluated by the CCK-8 assay. Data are presented as the means ± SD (n = 5, each group). ^**^P < 0.01 vs. *FUS*(−)NC + *circ_002136*(−)NC group. ^##^*P* < 0.01 vs. *FUS*(−) + *circ_002136*(−)NC group. (F) The co-effects of *FUS* and *circ_002136* on the migration of GECs were evaluated by the transwell assay. Data are presented as the means ± SD (n = 5, each group). ^**^P < 0.01 vs. *FUS*(−)NC + *circ_002136*(−)NC group. ^##^*P* < 0.01 vs. *FUS*(−) + *circ_002136*(−)NC group. The scale bar represents 100 μm. (G) The co-effects of *FUS* and *circ_002136* on the tube formation of GECs were evaluated by the Matrigel tube formation assay. Data are presented as the means ± SD (n = 5, each group). ^**^P < 0.01 vs. *FUS*(−)NC + *circ_002136*(−)NC group. ^##^*P* < 0.01 vs. *FUS*(−) + *circ_002136*(−)NC group. The scale bar represents 100 μm. (TIF 1639 kb)

## Abbreviations

3’-UTR: 3′-untranslated region
CCK-8: Cell counting kit-8
CDK11A: Cyclin-dependent kinase 11A
ChIP: Chromatin immunoprecipitation
circRNA: Circular RNA
EC: Endothelial cell
FUS: Fused in sarcoma
GBM: Glioblastoma
GEC: Glioma-exposed endothelial cells
HEK293T: Human embryonic kidney 293 T
miRNA: MicroRNA
NC: Negative control
qRT-PCR: Quantitative real-time PCR
RBP: RNA-binding protein
RISC: RNA-induced silencing complex
RNA-IP: RNA-binding protein immunoprecipitation
TSS: Transcription start site
