# Supplementary figures and images for: FUS/circ_002136/miR-138-5p/SOX13 feedback loop regulates angiogenesis in Glioma
Source: J Exp Clin Cancer Res. 2019 Feb 8;38:65. doi: 10.1186/s13046-019-1065-7 (PMC6368736; doi:10.1186/s13046-019-1065-7)

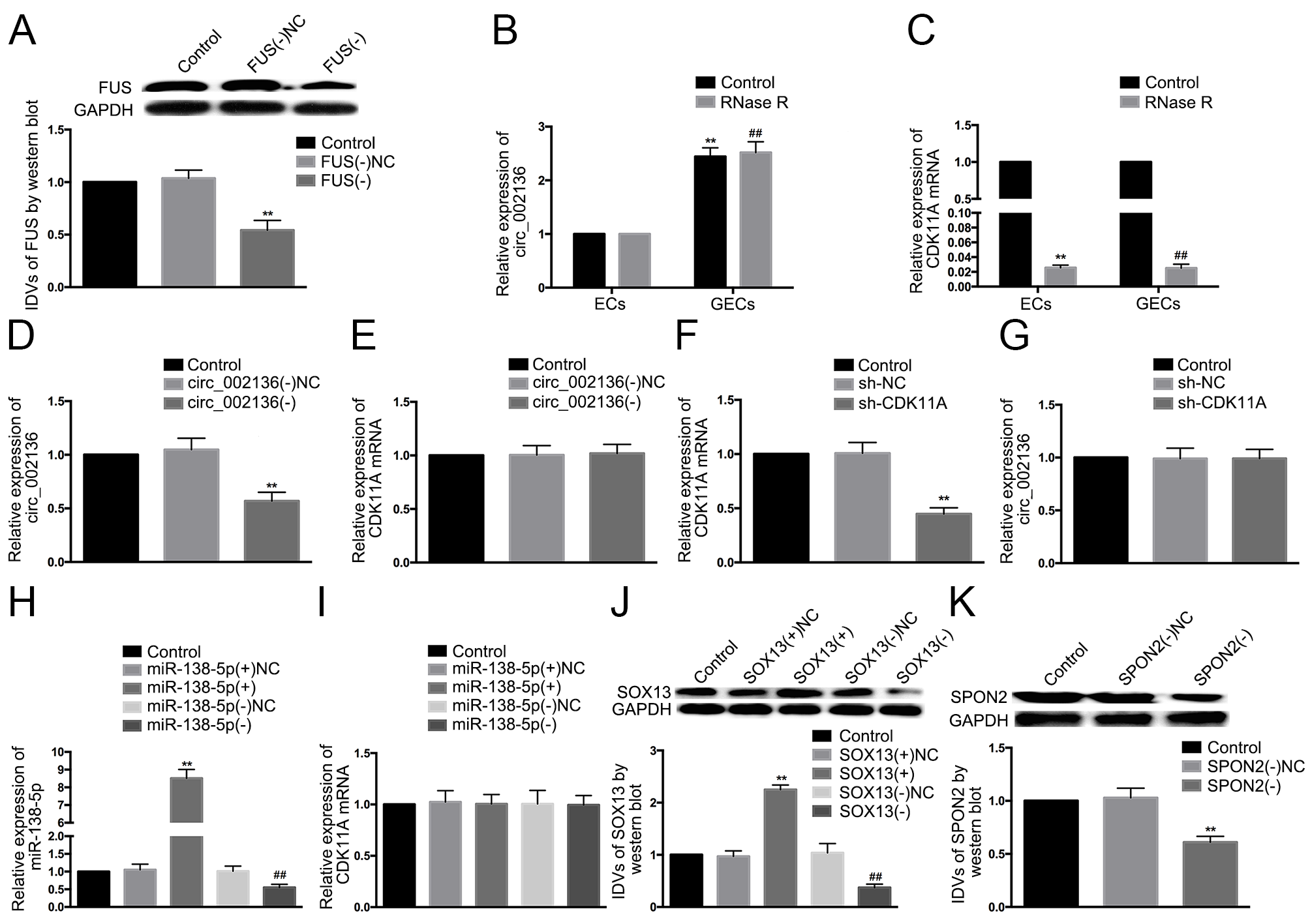

Supplement: Supplementary file 1 — Figure S1. Correlation between circ_002136, miR-138-5p and linear CDK11A and evaluation of the transfection efficiency. (A) The transfection efficiency of FUS. Data are presented as the means ± SD (n = 3, each group). **P < 0.01 vs. FUS (−) NC group. (B) The expression of circ_002136 with RNase R treatment. Data are presented as the means ± SD (n = 5, each group). **P < 0.01 vs. control group in ECs; ##P < 0.01 vs. RNase R group in ECs. (C) The expression of CDK11A with RNase R treatment. Data are presented as the means ± SD (n = 5, each group). **P < 0.01 vs. control group in ECs; ##P < 0.01 vs. control group in ECs. (D) The transfection efficiency of circ_002136 knockdown. Data are presented as the means ± SD (n = 5, each group). **P < 0.01 vs. circ_002136 (−) NC group. (E) The expression of CDK11A after circ_002136 knockdown. Data are presented as the means ± SD (n = 5, each group). (F) The transfection efficiency of CDK11A knockdown. Data are presented as the means ± SD (n = 5, each group). **P < 0.01 vs. sh-NC group. (G) The expression of circ_002136 after CDK11A knockdown. Data are presented as the means ± SD (n = 5, each group). (H) The transfection efficiency of miR-138-5p agomir or antagomir. Data are presented as the means ± SD (n = 5, each group). **P < 0.01 vs. miR-138-5p (+) NC group, ##P < 0.01 vs. miR-138-5p (−) NC group. (I) The expression of CDK11A after miR-138-5p over-expression or silencing. Data are presented as the means ± SD (n = 5, each group). (J) The transfection efficiency of SOX13. Data are presented as the means ± SD (n = 3, each group). **P < 0.01 vs. SOX13 (+) NC group, ##P < 0.01 vs. SOX13 (−) NC group. (K) The transfection efficiency of SPON2. Data are presented as the means ± SD (n = 3, each group). **P < 0.01 vs. SPON2 (−) NC group. (TIF 471 kb) [file 13046_2019_1065_MOESM1_ESM.tif]

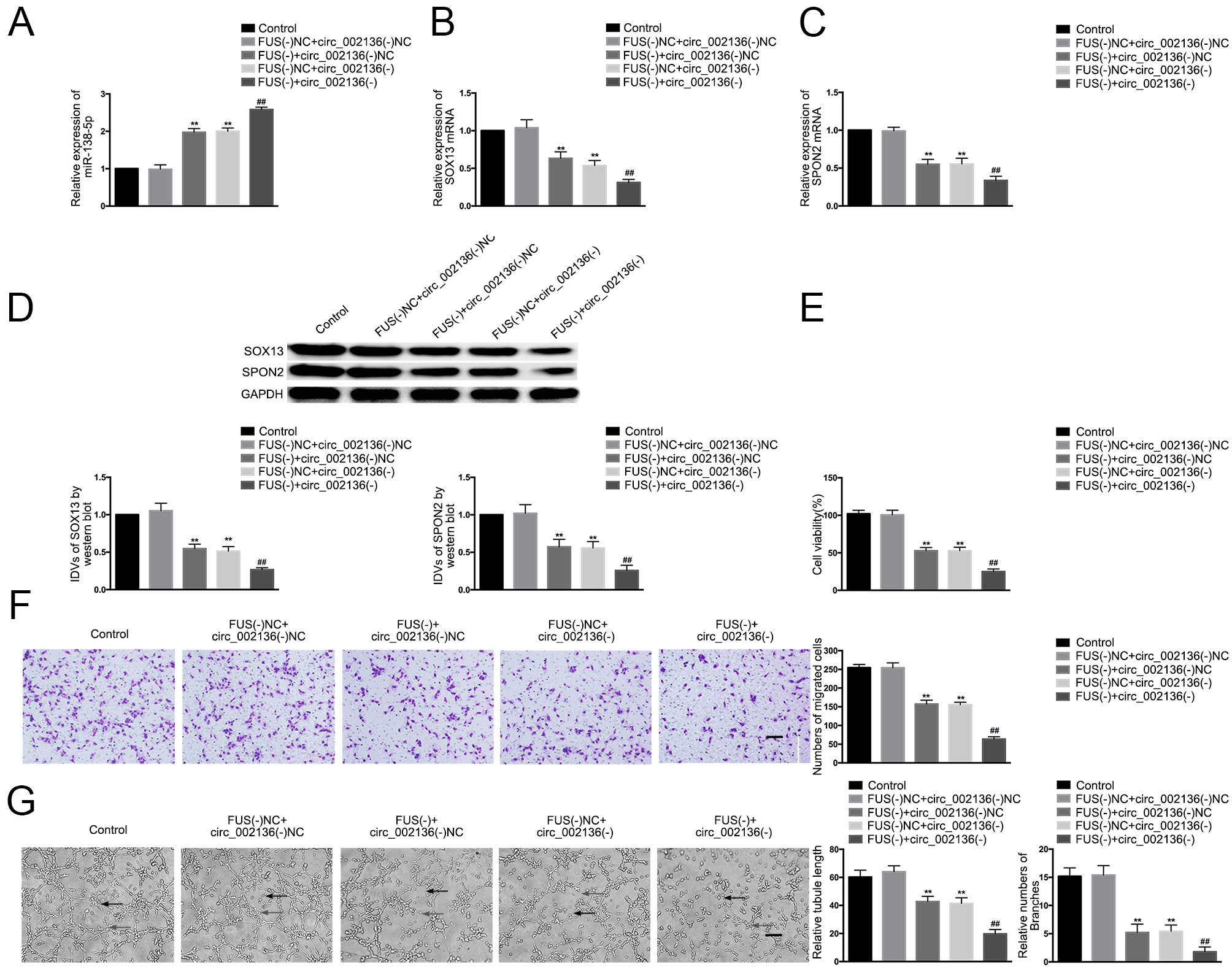

Supplement: Supplementary file 2 — Figure S2. Co-effects of FUS and circ_002136 on the expression of downstream molecules and angiogenesis. (A) The expression of miR-138-5p was co-regulated by both FUS and circ_002136. Values represent the means ± SD (n = 5, each group). **P < 0.01 vs. FUS(−)NC + circ_002136(−)NC group. ##P < 0.01 vs. FUS (−) + circ_002136(−)NC group. (B-D) The co-effects of FUS and circ_002136 on the mRNA and protein expression levels of SOX13 and SPON2 in GECs were evaluated by qRT-PCR and western blot. Data are presented as the means ± SD (n = 3, each group). **P < 0.01 vs. FUS(−)NC + circ_002136(−)NC group. ##P < 0.01 vs. FUS(−) + circ_002136(−)NC group. (E) The co-effects of FUS and circ_002136 on the viability of GECs were evaluated by the CCK-8 assay. Data are presented as the means ± SD (n = 5, each group). **P < 0.01 vs. FUS(−)NC + circ_002136(−)NC group. ##P < 0.01 vs. FUS(−) + circ_002136(−)NC group. (F) The co-effects of FUS and circ_002136 on the migration of GECs were evaluated by the transwell assay. Data are presented as the means ± SD (n = 5, each group). **P < 0.01 vs. FUS(−)NC + circ_002136(−)NC group. ##P < 0.01 vs. FUS(−) + circ_002136(−)NC group. The scale bar represents 100 μm. (G) The co-effects of FUS and circ_002136 on the tube formation of GECs were evaluated by the Matrigel tube formation assay. Data are presented as the means ± SD (n = 5, each group). **P < 0.01 vs. FUS(−)NC + circ_002136(−)NC group. ##P < 0.01 vs. FUS(−) + circ_002136(−)NC group. The scale bar represents 100 μm. (TIF 1639 kb) [file 13046_2019_1065_MOESM2_ESM.tif]
